# Interventions to improve team effectiveness within health care: a systematic review of the past decade

**DOI:** 10.1186/s12960-019-0411-3

**Published:** 2020-01-08

**Authors:** Martina Buljac-Samardzic, Kirti D. Doekhie, Jeroen D. H. van Wijngaarden

**Affiliations:** 1 Erasmus School of Health Policy & Management, Erasmus University Rotterdam, Bayle building, p.o. box 1738, 3000 DR Rotterdam, The Netherlands; 2 Erasmus School of Health Policy & Management, Erasmus University Rotterdam, Bayle building, p.o. box 1738, 3000 DR Rotterdam, The Netherlands; 3 Erasmus School of Health Policy & Management, Erasmus University Rotterdam, Bayle building, p.o. box 1738, 3000 DR Rotterdam, The Netherlands

**Keywords:** Systematic review, Healthcare teams, Intervention, Team training, Team tool, Team effectiveness, Team performance

## Abstract

**Background:**

A high variety of team interventions aims to improve team performance outcomes. In 2008, we conducted a systematic review to provide an overview of the scientific studies focused on these interventions. However, over the past decade, the literature on team interventions has rapidly evolved. An updated overview is therefore required, and it will focus on all possible team interventions without restrictions to a type of intervention, setting, or research design.

**Objectives:**

To review the literature from the past decade on interventions with the goal of improving team effectiveness within healthcare organizations and identify the “evidence base” levels of the research.

**Methods:**

Seven major databases were systematically searched for relevant articles published between 2008 and July 2018. Of the original search yield of 6025 studies, 297 studies met the inclusion criteria according to three independent authors and were subsequently included for analysis. The Grading of Recommendations, Assessment, Development, and Evaluation Scale was used to assess the level of empirical evidence.

**Results:**

Three types of interventions were distinguished: (1) *Training*, which is sub-divided into training that is based on predefined principles (i.e. CRM: crew resource management and TeamSTEPPS: Team Strategies and Tools to Enhance Performance and Patient Safety), on a specific method (i.e. simulation), or on general team training. (2) *Tools* covers tools that structure (i.e. SBAR: Situation, Background, Assessment, and Recommendation, (de)briefing checklists, and rounds), facilitate (through communication technology), or trigger (through monitoring and feedback) teamwork. (3) *Organizational (re)design* is about (re)designing structures to stimulate team processes and team functioning*.* (4) A *programme* is a combination of the previous types. The majority of studies evaluated a training focused on the (acute) hospital care setting. Most of the evaluated interventions focused on improving non-technical skills and provided evidence of improvements.

**Conclusion:**

Over the last decade, the number of studies on team interventions has increased exponentially. At the same time, research tends to focus on certain interventions, settings, and/or outcomes. Principle-based training (i.e. CRM and TeamSTEPPS) and simulation-based training seem to provide the greatest opportunities for reaching the improvement goals in team functioning.

## Introduction

Teamwork is essential for providing care and is therefore prominent in healthcare organizations. A lack of teamwork is often identified as a primary point of vulnerability for quality and safety of care [1, 2]. Improving teamwork has therefore received top priority. There is a strong belief that effectiveness of healthcare teams can be improved by team interventions, as a wide range of studies have shown a positive effect of team interventions on performance outcomes (e.g. effectiveness, patient safety, efficiency) within diverse healthcare setting (e.g. operating theatre, intensive care unit, or nursing homes) [3–7].

In light of the promising effects of team interventions on team performance and care delivery, many scholars and practitioners evaluated numerous interventions. A decade ago (2008), we conducted a systematic review with the aim of providing an overview of interventions to improve team effectiveness [8]. This review showed a high variety of team interventions in terms of type of intervention (i.e. simulation training, crew resource management (CRM) training, interprofessional training, general team training, practical tools, and organizational interventions), type of teams (e.g. multi-, mono-, and interdisciplinary), type of healthcare setting (e.g. hospital, elderly care, mental health, and primary care), and quality of evidence [8]. From 2008 onward, the literature on team interventions rapidly evolved, which is evident from the number of literature reviews focusing on specific types of interventions. For example, in 2016, Hughes et al. [3] published a meta-analysis demonstrating that team training is associated with teamwork and organizational performance and has a strong potential for improving patient outcomes and patient health. In 2016, Murphy et al. [4] published a systematic review, which showed that simulation-based team training is an effective method to train a specific type of team (i.e. resuscitation teams) in the management of crisis scenarios and has the potential to improve team performance. In 2014, O’Dea et al. [9] showed with their meta-analysis that CRM training (a type of team intervention) has a strong effect on knowledge and behaviour in acute care settings (as a specific healthcare setting). In addition to the aforementioned reviews, a dozen additional literature reviews that focus on the relationship between (a specific type of) team interventions and team performance could be mentioned [7, 10–19]. In sum, the extensive empirical evidence shows that team performance can be improved through diverse team interventions.

However, each of the previously mentioned literature reviews had a narrow scope, only partly answering the much broader question of how to improve team effectiveness within healthcare organizations. Some of these reviews focus on a specific team intervention, while others on a specific area of health care. For example, Tan et al. [7] presented an overview on team simulation in the operating theatre and O’Dea et al. [9] focused on CRM intervention in acute care. Other reviews only include studies with a certain design. For instance, Fung et al. [13] included only randomized controlled trials, quasi-randomized controlled trials, controlled before-after studies, or interrupted time series. Since the publication of our systematic review in 2010 [8], there has been no updated overview of the wide range of team interventions without restrictions regarding the type of team intervention, healthcare setting, type of team, or research design. Based on the number and variety of literature reviews conducted in recent years, we can state that knowledge on how to improve team effectiveness (and related outcomes) has progressed quickly, but at the same time is quite scattered. An updated systematic review covering the past decade is therefore relevant.

The purpose of this study is to answer two research questions: (1) What types of interventions to improve team effectiveness (or related outcomes) in health care have been researched empirically, for which setting, and for which outcomes (in the last decade)? (2) To what extent are these findings evidence based?

## Methodology

### Search strategy

The search strategy was developed with the assistance of a research librarian from a medical library who specializes in designing systematic reviews. The search combined keywords from four areas: (1) *team* (e.g. team, teamwork), (2) *health care* (e.g. health care, nurse, medical, doctor, paramedic), (3) *interventions* (e.g. programme, intervention, training, tool, checklist, team building), (4) *improving team functioning* (e.g. outcome, performance, function) OR a specific *performance outcome* (e.g. communication, competence, skill, efficiency, productivity, effectiveness, innovation, satisfaction, well-being, knowledge, attitude). This is similar to the search terms in the initial systematic review [8]. The search was conducted in the following databases: EMBASE, MEDLINE Ovid, Web of Science, Cochrane Library, PsycINFO, CINAHL EBSCO, and Google Scholar. The EMBASE version of the detailed strategy was used as the basis for the other search strategies and is provided as additional material (see Additional file 1). The searches were restricted to articles published in English in peer-reviewed journals between 2008 and July 2018. This resulted in 5763 articles. In addition, 262 articles were identified through the systematic reviews published in the last decade [3, 4, 7, 9–28]. In total, 6025 articles were screened.

### Inclusion and exclusion criteria

This systematic review aims to capture the full spectrum of studies that *empirically* demonstrate how *healthcare* organizations could *improve* team effectiveness. Therefore, the following studies were excluded:

1. Studies outside the healthcare setting were excluded. Dental care was excluded. We did not restrict the review to any other healthcare setting.
2. Studies without (unique) empirical data were excluded, such as literature reviews and editorial letters. Studies were included regardless of their study design as long as empirical data was presented. Book chapters were excluded, as they are not published in peer-reviewed journals.
3. Studies were excluded that present empirical data but without an outcome measure related to team functioning and team effectiveness. For example, a study that evaluates a team training without showing its effect on team functioning (or care provision) was excluded because it does not provide evidence on how this team training affects team functioning.
4. Studies were excluded that did not include a team intervention or that included an intervention that did not primarily focus on improving team processes, which is likely to enhance team effectiveness (or other related outcomes). An example of an excluded study is a training that aims to improve technical skills such as reanimation skills within a team and sequentially improves communication (without aiming to improve communication). It is not realistic that healthcare organizations will implement this training in order to improve team communication. Interventions in order to improve collaboration between teams from different organizations were also eliminated.
5. Studies with students as the main target group. An example of an excluded study is a curriculum on teamwork for medical students as a part of the medical training, which has an effect on collaboration. This is outside the scope of our review, which focuses on how healthcare *organizations* are able to improve team effectiveness.

In addition, how teams were defined was not a selection criterion. Given the variety of teams in the healthcare field, we found it acceptable if studies claim that the setting consists of healthcare teams.

### Selection process

**Fig. 1:**
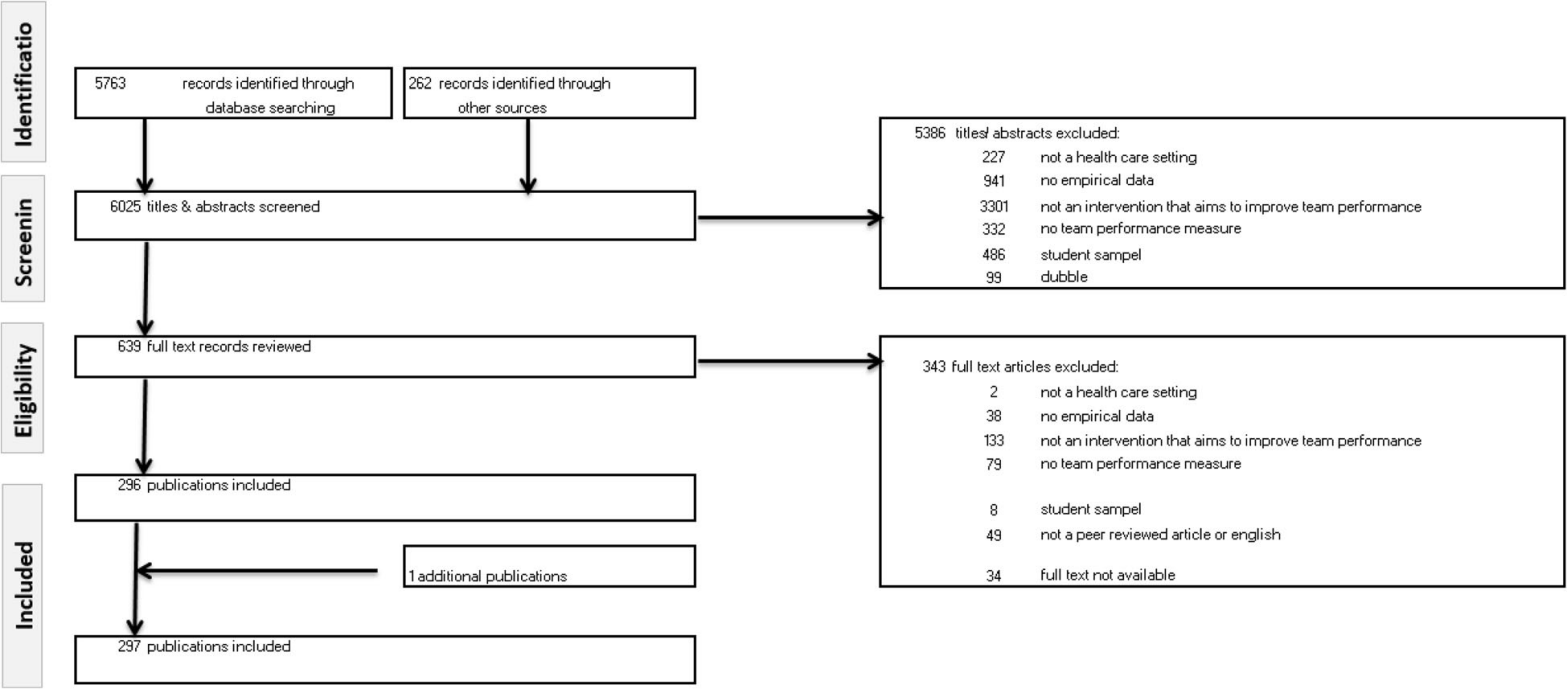
PRISMA flowchart

Figure 1 summarizes the search and screening process according to the Preferred Reporting Items for Systematic Reviews and Meta-Analyses (PRISMA) format. A four-stage process was followed to select potential articles. We started with 6025 articles. First, each title and abstract was subjected to elimination based on the aforementioned inclusion and exclusion criteria. Two reviewers reviewed the title/abstracts independently. Disagreement between the reviewers was settled by a third reviewer. In case of doubt, it was referred to the next stage. The first stage reduced the number of hits to 639. Second, the full text articles were assessed for eligibility according to the same set of elimination criteria. After the full texts were read by two reviewers, 343 articles were excluded. In total, 297 articles were included in this review. Fourth, the included articles are summarized in Table 1. Each article is described using the following structure:

- Type of intervention
- Setting: the setting where the intervention is introduced is described in accordance with the article, without further categorization
- Outcomes: the effect of the intervention
- Quality of evidence: the level of empirical evidence is based in the Grading of Recommendations Assessment Development, and Evaluation (GRADE) scale. GRADE distinguishes four levels of quality of evidence
  A. High: future research is highly unlikely to change the confidence in the estimated effect of the intervention.
  B. Moderate: future research is likely to have an important impact on the confidence in the estimated effect of the intervention and may change it.
  C. Low: future research is very likely to have an important impact on the confidence in the estimated effect of the intervention and is likely to change it.
  D. Very low: any estimated effect of the intervention is very uncertain.

**Table 1:** Summary of results

Authors (year)	Intervention	Setting	Outcome(s)	GRADE
Principle-based training: CRM-based training				
Allan et al. 2010 [29]	A simulation-based in situ CRM training: game play, didactics, video review, hands-on high-fidelity simulation-based training and video-based debriefing	Paediatric cardiac intensive care	Improvement in participants’ perceived ability to function as a code team member and confidence in a code, likeliness to raise concerns about inappropriate management to the code leader	C
Ballangrud et al. 2014 [30]	Simulation-based CRM team training: introductory theory inputs on safe team performance based on CRM and a team training in a simulation laboratory	Intensive care	Training increases awareness of clinical practice and acknowledges the importance of structured work in teams	D
Bank et al. 2014 [31]	Needs-based paediatric CRM simulation training with post activity follow-up: plenary educational session, simulation and debriefing	Paediatric emergency medicine residents (postgraduate year 1–5)	Improvement in the ability to be an effective team leader in general, delegating tasks appropriately, and ability to ensure closed loop communication, and identification of CRM errors	C
Budin et al. 2014 [32]	CRM training: train-the-trainer programme and CRM training including videos, lecture, and role playing	Perinatal care	Improvement in nurse and physician perceptions of teamwork and safety climate	C
Carbo et al. 2011 [33]	CRM-based training focusing on appropriate assertiveness, effective briefings, callback and verification, situational awareness, and shared mental models	Inpatient internal medicine	Improvement in the percentage of correct answers on a question related to key teamwork principles, reporting “would feel comfortable telling a senior clinician his/her plan was unsafe”	C
Catchpole et al. 2010 [34]	Aviation-style team training: classroom training of interactive modules including lectures and discussions, and coaching in theatre	Surgery	More time-outs, briefings, and debriefings	B
Clay-Williams et al. 2013 [35]	CRM-based classroom training, CRM simulation training or classroom training followed by simulation training	Doctors, nurses and midwives	Improvement in knowledge, self-assessed teamwork behaviour and independently observed teamwork behaviour when classroom-only trained group was compared with control, these changes were not found in the group that received classroom followed by simulation training	A
Cooper et al. 2008 [36]	Simulation-based anaesthesia CRM training	Anaesthesiology	No difference between the trained and untrained cohorts	C
France et al. 2008 [37]	CRM training: CRM introductory training course (i.e. lectures, case studies, and role playing) and perioperative CRM training (i.e. e-learning models and toolkit consisting of CRM process checklist, briefing scripts, communication whiteboard, implementation training)	Surgery	Shows potential to improve team behaviour and performance	D
Gardner et al. 2008 [38]	Simulation-based CRM training with debriefing	Obstetrics department	Reduction in annual obstetrical malpractice premiums; improvement in teamwork and communication in managing a critical obstetric event in the interval	C
Gore et al. 2010 [39]	CRM training: educational seminar (i.e. lectures and role-play exercises), development and expansion of time-out briefing, educational video on briefing, posters on content briefing	Operating room	Improvement in teamwork, error reporting, and safety climate	C
Haerkens et al. 2017 [40]	CRM training: CRM awareness training (i.e. lectures and multiple interactive sessions using case studies and video footage), implementation of tools	Emergency department	Improvement in teamwork climate, safety climate and stress recognition. Increase in patient length of stay	B
Haller et al. 2008 [41]	CRM training: video, discussion, (interactive) lectures, workshops, play roles, interactive course	Obstetrical setting in hospital	Improvement in knowledge of teamwork, shared decision making, team and safety climate, stress recognition	B
Hefner et al. 2017 [42]	CRM training: day-long retreats, during which participants underwent developed and tailored CRM safety tools and participated in role playing, development of system-wide internal monitoring processes	Medical centre consisting of multiple hospitals and two campuses	Improvement in (1) organizational learning and continuous improvement, (2) overall perceptions of patient safety, (3) feedback and communication about errors, and (4) communication openness.	B
Hicks et al. 2012 [43]	Crisis Resources for Emergency Workers (CREW): a simulation-based CRM curriculum: precourse learning and a full-day simulation-based exercise with debriefing	Emergency department	Believe that CREW could reduce errors and improve patient safety; no improvement toward team-based attitudes	C
Hughes et al. 2014 [44]	CRM adapted to Trauma Resuscitation with new cultural and process expectation: CRM course of 15 sessions	Trauma resuscitation	Improvement in accuracy of field to medical command information, accuracy of emergency department medical command information to the resuscitation area, team leader identity, communication of plan, role assignment, likeliness to speak up when patient safety was a concern	B
de Korne et al. 2014 [45]	Team Resource Management (TRM) programme (based on CRM concepts): safety audits of processes and (team) activities, interactive classroom training sessions by aviation experts, a flight simulator session, and video recording of team activities with subsequent feedback	Eye hospital	Observations suggests increase safety awareness and safety-related patterns of behaviour between professions, including communication	D
Kuy and Romero 2017 [46]	CRM training: didactics, group discussions, and simulation training	Surgical service staff at a VA Hospital	At T1 participants reported improvement in all 27 areas assessed. At T2 his improvement was sustained in 85% of the areas studied. Areas with largest improvement: briefing, collaboration, nursing input, and patient safety. Areas with regression: speaking up, expressing disagreement, level of staffing, and discussing errors	C
LaPoint et al. 2012 [47]	CRM training: core skills workshops	Perioperative staff	Improvement in supervisor expectations, communication openness, teamwork within units, non-punitive response to error, hospital management support for safety, handoffs. No significant improvement in organizational learning, feedback communication about errors, teamwork across hospital units, number of events	C
Mahramus et al. 2016 [48]	Teamwork training based on CRM and TeamSTEPPS: simulations, debriefing, teamwork education	Hospital	Improvement in perceptions of teamwork behaviours	C
McCulloch et al. 2009 [49]	Classroom non-technical skills training based on CRM: mixed didactic and interactive teaching (e.g. role play), follow-up feedback by trainers	Operating room	Improved technical and non-technical performance: improvement in attitudes to safety, team non-technical performance and technical error rates	C
Mehta et al. 2013 [50]	Multidisciplinary simulation course: CRM teaching, simulation with debriefing, closing session with feedback	Operating room	Improvement in clinical knowledge, teamwork, leadership and non-technical skills, as well as the mutual understanding and respect between related medical and non-medical team members	D
Morgan et al. 2015a [51]	CRM-based training and improving working processes through implementing morning briefing and WHO Surgical Safety Checklist	Operating room conducting elective orthopaedic surgery	Improvement in non-technical skills and WHO compliance; no significant improvement in clinical outcomes	C
Morgan et al. 2015b [52]	Teamwork training course CRM-based interactive classroom teaching and on the job coaching	Operating rooms	Improvement in non-technical skills, but also with a rise in operative glitches	B
Muller et al. 2009 [53]	CRM training (i.e. psychological teaching including theoretical exercises and simulator scenarios and video-assisted debriefing) versus classic simulator training (MED)	Hospital	Improvement in clinical and non-technical performance after both training, but no difference between training	C
Parsons et al. 2018 [54]	Simulation-based CRM training: didactic presentation, series of simulation scenarios and structured debriefs	Emergency medicine	No significant improvement in leadership, problem solving, communication, situational awareness, teamwork, resource utilization and overall CRM skills	D
Phipps et al. 2012 [55]	CRM-based training: didactic sessions, simulation and debriefing	Labour and delivery	Improvement in patient outcomes (adverse outcomes), perceptions of patient safety including the dimensions of teamwork and communication	B
Ricci et al. 2012 [56]	CRM training: Training (i.e. didactics, case study discussions, team-building exercises, simulated operating room brief and debrief sessions) and CRM techniques (e.g. pre-operative checklist and brief, post-operative debrief, read and initial files, feedback tools)	Perioperative personnel	Wrong site surgeries and retained foreign bodies decreased, but increased after 14 months without additional training.	B
Robertson et al. 2009 [57]	Obstetric Crisis Team Training: online module, training session (standardized, simulated crisis scenarios with simulator mannequin), and debriefings	Multidisciplinary obstetric providers in hospital	Improvement in attitude; perception of individual and team performance, and overall team performance	C
Savage et al. 2017 [58]	CRM safety programme: CRM training (i.e. didactic seminars, role playing), systematic risk assessments, and improving work practices (i.e. checklists, huddles or structured communication and meeting tools)	Paediatric surgery	Improvement in non-technical skills, the use of safety tools, adherence to guidelines, safety culture (i.e. teamwork across and within units, supervisors’ expectations and actions, non-punitive response to adverse events, perceptions of overall patient safety); unplanned readmissions following appendectomy declined	A
Sax et al. 2009 [59]	CRM training: video, team building exercises, open forum, and development and implementation of perioperative checklist	Hospitals	Increased self-initiated error reports and perceived self-empowerment	B
Shea-Lewis et al. 2009 [60]	CRM-based training: real-life examples, feedback, SBAR, team meetings, briefing, and debriefing	Obstetric department	Improvement in patient outcome, patient satisfaction, employee satisfaction	C
Schwartz et al. 2018 [61]	Clinical Team Training (CTT) based on CRM principles: training (e.g. simulation) and implementation of improvement projects (e.g. briefing, huddles, checklists)	Veterans Health Administration facilities	Improvement in communication, teamwork and situational awareness for patient safety. Also decreased between T1 and T2 detected.	B
Sculli et al. 2013 [62]	Nursing CRM: interactive didactic training curriculum, features high-fidelity simulation, ongoing consultation, improvement project, refreshment	Nursing units	Improvement unit climate, teamwork, medication errors, HAPU, glucose control, FTR events, and care processes	C
Steinemann et al. 2011 [63]	Crisis Team Training-based in situ team training: web-based didactic, simulations, and debriefing	Emergency department	Improvement in teamwork ratings, clinical task speed and completion rates, teamwork scores, objective parameters of speed and completeness of resuscitation	B
Stevens et al. 2012 [64]	CRM-based educational programme based on high realism acute crisis simulation scenarios and interactive workshop	Cardiac surgery	Survey: improvement in the concept of working as a team. Interview: improvement in personal behaviours and patient care, including speaking up more readily and communicating more clearly	D
Suva et al. 2012 [65]	CRM training: introductory course, interactive workshops, lecture, role play	Operating room	Improvement in learning, knowledge regarding teamwork, safety climate, and stress recognition; improvement varies with participant specialty	C
Tschannen et al. 2015 [66]	Nursing CRM training: educational sessions, podcasts, simulation and debriefing	General medicine telemetry unit	No significant improvement in communication openness and environmental values; RNs reported an increase in both synchronous communication and asynchronous communication with physicians whereas physicians noted a reduction in time spent in asynchronous communication	D
West et al. 2012 [67]	Nursing CRM training: didactic session, simulation, implementation of a CRM technique: sterile cockpit rule	Veterans Affairs hospital on nursing units	Improvement in efficiency (e.g. quicker follow-up on abnormal vital signs and blood glucose levels, rapid assessment of patients with changes in condition, and faster intervention when the condition was deteriorating) and perceived teamwork, communication, patient safety	C
Ziesmann et al. 2013 [68]	STARTT (Standardized Trauma and Resuscitation Team Training): lectures (on CRM), discussion based on CRM principles, simulations and debriefing	Trauma teams	Improvement in overall CRM domains, teamwork, and safety climate	D
Principle-based training: TeamSTEPPS				
Armour Forse et al. 2011 [69]	TeamSTEPPS	Operating room	Improvement in communications, leadership first case starts, Surgical Quality Improvement Program measures, surgical morbidity and mortality, culture; not all improvement were sustained. No significant effect on PACU communication and teamwork	B
Bridges et al. 2014 [70]	Educational intervention: adapted TeamSTEPPS curriculum, discussion, practicing standardized communication tools	Hospital Intermediate Care Unit serving adult medical cardiac patients	Improvement in awareness of teamwork and backup	C
Brodsky et al. 2013 [71]	Multidisciplinary, small group, interactive workshop based on TeamSTEPPS	Neonatal intensive care	Improvement in the overall teamwork, communication, situation awareness, support, satisfaction, job fulfilment, respect	B
Bui et al. 2018 [72]	Video and live observation of TeamSTEPPS skills implementation during surgical briefs and debriefs	Operating rooms	Low compliance with TeamSTEPPS skills; compliance was under video observation than under live observation	D
Capella et al. 2010 [73]	TeamSTEPPS (e.g. didactic session, simulation, 5 tools: briefing, STEP (situation monitoring tool), CUS (mutual support tool), call outs, and check backs)	Level I trauma centre	Improvement in leadership situation monitoring, mutual support, communication, and overall teamwork; decreasing the times from arrival to the CT scanner, endotracheal intubation and the operating room	B
Castner et al. 2012 [74]	TeamSTEPPS	Hospital inpatient bedside RNs	Improved perceptions of leadership	C
Deering et al. 2011 [75]	TeamSTEPPS	Combat support hospital	Decreases in the rates of communication-related errors, medication and transfusion errors, and needles tick incidents, the rate of incidents coded communication as the primary teamwork skill that could have potentially prevented the event	C
Figueroa et al. 2013 [76]	TeamSTEPPS-based simulation training: lecture (on TeamSTEPPS principles), simulation, checklist, and debriefing	Paediatric cardiovascular intensive care	Improving confidence, skills in the role of team leaders, and TeamSTEPPS concepts	B
Gaston et al. 2016 [77]	Customized TeamSTEPPS training (of 2 instead of 6 h)	Oncology acute patient care	Improvement in staff perception of teamwork and communication	B
Gupta et al. 2015 [78]	A selection of TeamSTEPPS tools	Academic interventional ultrasound service	Improvement in teamwork climate, safety climate, and teamwork	C
Harvey et al. 2014 [79]	In situ simulation-based training (SBT) versus case study review, both incorporating TeamSTEPPS training	Medical-surgical PCUs	Improvement in knowledge and teamwork skills in both groups; SBT group showed greater improvement in all areas except knowledge	C
Jones et al. 2013 [80]	TeamSTEPPS (e.g. TeamSTEPPS tools, fundamentals course)	Hospitals	Improvement in safety culture	A
Jones et al. 2013 [81]	TeamSTEPPS (e.g. essentials course)	Emergency department	Improvement of staff perception related to a culture of safety (e.g. management support for patient safety, feedback and communications about error, communication openness)	B
Lee et al. 2017 [82]	After TeamSTEPPS, implementation of reinforcement activities regarding leadership and communication (i.e. lectures, self-paced learning programme, 1 page summary, and grand rounds on TeamSTEPPS principles)	Orthopaedic surgery	Nursing staff: improvement in leadership and communication behaviours. Surgical staff: improvement in leadership behaviours. Anaesthesia staff: no improvement in any teamwork behaviours	C
Lisbon et al. 2016 [83]	TeamSTEPPS: brief, huddle, DESC (constructive approach for managing and resolving Conflict) and CUS script	Academic emergency department	Improvement in knowledge and improved communication attitudes; adoption of a specific behaviour, the huddle, also was observed	B
Mahoney et al. 2012 [84]	TeamSTEPPS (variation of tools: flyers, simulations, games, and sustainment tools such as luncheon debriefing, awards)	Psychiatric hospital	Improvement in team foundation, functioning, performance, skills, climate, and atmosphere	B
Mayer et al. 2011 [85]	TeamSTEPPS (e.g. fundamental curriculum)	Paediatric and surgical intensive care	Improvement in experienced teamwork, team performance, communication openness and clinical outcomes (e.g. average time for placing patients on extracorporeal membrane oxygenation, average duration of adult surgery rapid response team events	B
Rice et al. 2016 [86]	Modified simulation-based TeamSTEPPS training	Intensive care	Improvement in teamwork attitudes, perceptions, and performance	D
Riley et al. 2011 [87]	TeamSTEPPS didactic training (e.g. webinar, video of simulated scenarios) versus full TeamSTEPPS training (e.g. series of in situ simulation training exercises including (de)briefing, rapid-cycle follow-through with process improvements, and repetition	Hospitals	Improvement in perinatal morbidity between the pre- and post-intervention for hospital with simulation programme. No significant changes in safety culture	B
Sawyer et al. 2013 [88]	TeamSTEPPS training (e.g. fundamental course) with medical simulation	Neonatal intensive care	Improvement in teamwork skills in team structure, leadership, situation monitoring, mutual support, and communication, the odds of a nurse challenging an incorrect medication dose, and detection and correction of inadequate chest compressions	C
Sonesh et al. 2015 [89]	Adapted TeamSTEPPS (lecture-based interactive programme)	Obstetrical setting	Improvement in knowledge of communication strategies, decision accuracy, and length of babies’ hospital length of stay. Knowledge of other team competencies or self-reported teamwork did not significantly improve	C
Spiva et al. 2014 [90]	Training curriculum based on TeamSTEPPS (e.g. didactic lecture, patient video vignettes, debriefing)	Hospital	Improvement on fall reduction and teamwork	B
Stead et al. 2009 [91]	TeamSTEPPS (e.g. redesign meetings, SBAR, coaching)	Mental health facility	Substantial impact on patient safety culture (i.e. frequency of event reporting, and curriculum learning), teamwork, communication, KSA score, rates of seclusion. Issues around staffing, teamwork across hospital units, and hospital management support remained unchanged	D
Thomas et al. 2012 [92]	TeamSTEPPS (e.g. master trainer course, fundamentals course, essentials course)	Hospital	Improvement in feedback and communication about error, frequency of events reported, hospital handoff and transitions, staffing, and teamwork across the units	C
Treadwell et al. 2015 [93]	TeamSTEPPS (e.g. huddle, debrief, SBAR, briefing checklist)	Medical home	Improved perception of team collaboration	C
Vertino 2014 [94]	TeamSTEPPS (e.g. formal presentation, discussion, role-play exercises embodying clinical scenarios)	Inpatient (VHA) hospital unit	Positive change in staff attitudes toward team structure, leadership, situation monitoring, mutual support, and communication	D
Weaver et al. 2010 [15]	TeamSTEPPS (e.g. didactic session, interactive role playing, multiple tools)	Operating rooms	Improvement in quality and quantity of briefings and the use of quality teamwork behaviours during cases	B
Wong et al. 2016 [95]	Interprofessional education course: adapted TeamSTEPPS curriculum, simulation scenarios, and structured debriefing, and wrap-up session	Emergency department	Improvement in team structure, leadership, situation monitoring, mutual support, frequency of event reporting, teamwork within hospital units, and hospital handoffs and transitions	B
Method-based training: Simulation-based training				
AbdelFattah et al. 2018 [96]	Trauma-focus simulation training: trauma simulations with video-based debriefing	Trauma surgery	Improvement in clinical management, leadership, communication, cooperation, professionalism and performance on trauma rotation	D
Amiel et al. 2016 [97]	One-day simulation- based training with video-based debriefing	Emergency department in trauma centre	Improvement in teamwork, communication, patient handoff, and shock and haemorrhage control	C
Arora et al. 2014 [98]	Full-hospital simulation across the entire patient pathway (with integration of teams in prehospital, through-hospital, and post-hospital care)	Hospital	Improvement in decision making, situational awareness, trauma care, and knowledge of hospital environment. Behavioural skills, such as teamwork and communication, did not show significant improvement	C
Arora et al. 2015 [99]	Simulation-based training for improving residents’ management of post-operative complications: ward-based scenarios and debriefing intervention	Surgery	Clinically, improvement in residents’ ability to recognize/respond to falling saturations, check circulatory status, continuously reassess patient, and call for help. Teamwork, improvement in residents’ communication, leadership, decision-making skills, and interaction with patients (empathy, organization, and verbal and nonverbal expression)	B
Artyomenko et al. 2017 [100]	Simulation training sessions for urgent conditions with debriefing	Obstetrical anaesthesiologists	Improvement in speed and invasive techniques, teamwork and effectiveness after the fifth session	C
Auerbach et al. 2014 [101]	In situ interdisciplinary paediatric trauma quality improvement simulation: simulated patient care followed by debriefing	Tertiary care paediatric emergency department	Improvement in overall performance, teamwork, and intubation subcomponents	C
Bender et al. 2014 [102]	Simulation-enhanced booster session (after Neonatal Resuscitation Program): orientations session, simulation, and debriefing	Paediatric and Family Practice	The intervention group demonstrated better procedural skills and teamwork behaviours. The NICU programme demonstrated better teamwork behaviours compared with non-NICU programme	B
Bittencourt et al. 2015 [103]	In centre simulation-based training (simulation and debriefing) and in situ simulation (simulation and debriefing): comparison of actual paediatric emergencies, in-centre simulations, and in situ simulations	Paediatric level 1 trauma centre	Mean total TEAM scores were similar among the 3 settings. Simulation-based training improved communication, team interaction, shared mental models, clarifying roles and responsibilities, and task management	B
Bruppacher et al. 2010 [104]	Training session with either high-fidelity simulation-based training (i.e. orientation session, simulation, and debriefing) or an interactive seminar (i.e. audiovisual aids such as PowerPoint slides, handouts, and face-to-face discussion of paper-based scenarios similar to the simulation training)	Anaesthesiology for cardiopulmonary bypass	Both groups improved, the simulation group showed significantly higher improvement on situation awareness, team working, decision making, task management, and checklist performance compared with the seminar group	B
Bursiek et al. 2017 [105]	Interdisciplinary (high-fidelity) simulation training with debriefing	Interdisciplinary teams	Improvement in team work, perception of work environment and patient safety	C
Burton et al. 2011 [106]	Simulation-based training: simulation laboratory curriculum with video-assisted debriefings	Extracorporeal membrane oxygenation emergencies	No improvement in timed responses or percent correct actions. Improvement in teamwork, knowledge, and attitudes	C
Chung et al. 2011 [107]	Conventional simulation-based training (i.e. lecture, videos, simulations, and debriefing) versus a script-based training	Cardiopulmonary resuscitation in emergency departments	Both type of training improved leadership scores, but no improvement in performance	B
Cooper et al. 2012 [108]	Simulation team training: formative questionnaire, team-based videoed scenarios, photo elicitation, and expert feedback sessions	Hospital nurse teams	Improvement in knowledge, confidence and competence; group debriefing session enhanced learning	C
Ciporen et al. 2018 [109]	Crisis management simulation training: instructions, simulation, and debriefing	Neurosurgery and anaesthesiology	No significant differences between groups in situation awareness, decision making, communication and teamwork	C
Ellis et al. 2008 [110]	High-technology training at a simulation centre versus low-tech training in local units (with and without teamwork theory)	Midwives and obstetricians in hospitals	Improvement in rates of completion for basic tasks, time to administration of magnesium sulphate, and teamwork. Training in a simulation centre and teamwork theory had no effect	B
Fernando et al. 2017 [111]	Interprofessional simulation training with debriefing	Primary and secondary care doctors	Improvement in knowledge, confidence and attitudes. Qualitative data indicates improvement in clinical skills, reflective practice, leadership, teamwork and communication skills	C
Fouilloux et al. 2014 [112]	Training based on an animal simulation model	Cardiac surgery	Improvement in management of the adverse events and time spend per certain events	D
Fransen et al. 2012 [113]	Multiprofessional simulation team training: introduction video, simulation, and debriefing	Obstetric departments	Improvement in teamwork performance and use of the predefined obstetric procedures	A
Freeth et al. 2009 [114]	Simulation-based interprofessional training with video-recorded debriefing	Delivery	Improvement in knowledge and understanding of interprofessional team working, especially communication and leadership in obstetric crisis situations	C
Frengley et al. 2011 [115]	Simulation-based training: familiarization, teamwork session (presentation, video, and discussions), skills station, simulations or case-based training	Critical care	Improvement in overall teamwork, leadership, team coordination, verbalizing situational information, clinical management; no difference between simulation-based learning and case-based learning	B
George and Quatrara 2018 [116]	Interprofessional simulation training: introduction session, simulation, and debriefing	Surgical trauma burn intensive care unit	Improvement in perceptions of teamwork and knowledge	D
Gettman et al. 2009 [117]	High-Fidelity Operating Room Simulation: introduction, simulation, and video-based debriefing	Orology, operating room	Improvement in teamwork, communication, laparoscopic skills, and team performance	C
Gilfoyle et al. 2017 [118]	Simulation-based training: lecture, group discussions, simulations, and debriefing	Paediatric resuscitation	Improvement in clinical performance and clinical teamwork (role responsibility, communication, situational awareness and decision making)	B
Gum et al. 2010 [119]	Interprofessional simulation training with video-based debriefing	Maternity emergency	Ability for collaboration in team building (i.e. personal Role Awareness, interpositional knowledge, mutuality and leadership)	D
Hamilton et al. 2012 [120]	High-fidelity simulated trauma resuscitation with video-assisted debriefing	Surgery	Improvement in team function score and the feeling of being more competent as team leaders and team members	B
Hoang et al. 2016 [121]	Training course: classroom didactic sessions and hand-on simulation sessions	(U.S. Navy Fleet) surgery	Improvement in time to disposition and critical errors	D
James et al. 2016 [122]	Simulation-based interprofessional team training: simulation followed by debriefing and performance feedback	Oncology	Acquired new knowledge, skills, and attitudes to enhance interprofessional collaboration	C
Kalisch et al. 2015 [123]	Virtual simulation training with introduction session	Medical–surgical patient care unit	Improvement in overall teamwork, trust, team orientation, and backup	D
Khobrani et al. 2018 [124]	Boot camp curriculum with high-fidelity paediatric simulations with debriefing	(Paediatric) emergency medicine	Improvement in teamwork performance (leadership, cooperation, communication, assessment and situation) and basic knowledge	D
Kilday et al. 2012 [125]	Team intervention: didactic curriculum with skill lab practice sessions, simulations, debriefing	Hospitals	Improvement in team performance, knowledge, and emergency teamwork	C
Kirschbaum et al. 2012 [126]	Multidisciplinary team training: assessments, high-fidelity simulation sessions, and debriefing	Obstetricians and anaesthesiologists	Improvement in teamwork cultural attitudes and perceptions, communication climate; decreases in autonomous cultural attitudes and perceptions	C
Koutantji et al. 2008 [127]	Simulations with debriefing and in between an interactive workshop on briefing, check-listing methods and protocol	Surgery	Improvement in technical skills and no or negative effect on non-technical skills	D
Kumar et al. 2018 [128]	Simulation-based Practical Obstetric Multi-Professional Training (PROMPT): interactive lectures, scenarios based drills, debriefing	Obstetric care in hospitals	Improvement in clinical and non-technical skills highlighting principles of teamwork, communication, leadership and prioritization in an emergency situation. No significant change in clinical outcomes	B
Larkin et al. 2010 [129]	Simulation-Based curriculum: video demonstrations, triggers, and simulated scenarios	Surgery	Improvement in empathic communication. Higher levels of stress. No significant improvement in teamwork attitudes	C
Lavelle et al. 2018 [130]	Multidisciplinary simulation-based training designed to address Medical Emergencies in Obstetrics: lecture, orientation session, simulation, debriefing, didactic teaching	Healthcare staff across organizations	Improvement in clinical skills and non-technical skills including teamwork, communication and leadership skills	D
Lavelle et al. 2017 [131]	In situ, simulation training: introduction, simulation, and debriefing	Psychiatric triage wards	Improvement in knowledge, confidence, and attitudes toward managing medical deterioration. Based on reflection: improved confidence in managing medical deterioration, better understanding of effective communication, improved self-reflection and team working, and an increased sense of responsibility for patients’ physical health. Incident reporting increased by 33%	C
Lee et al. 2012 [132]	Interdisciplinary high-fidelity simulation-based team training with debriefing	Urology	Urology resident training correlated with technical performance but not with non-technical performance; anaesthesia resident training level did correlate with non-technical performance	D
Lorello et al. 2016 [133]	Mental practice training (versus ATLS training) and simulation with debriefing	Trauma resuscitation	Improvement in teamwork behaviour, compared to traditional simulation-based trauma instruction	B
Mager et al. 2012 [134]	Expanded Learning and Dedication to Elders in the Region (ELDER): simulated patient scenarios using mid-fidelity human patient simulators and debriefing	Long-term care facilities and home care agency	Encouraging communication and teamwork	C
Maxson et al. 2011 [135]	Interdisciplinary simulation team training with high-fidelity simulation scenarios, pre- and debriefing session	Inpatient surgical ward	Improvement in collaboration between nurses and physicians and patient care decision making process	C
McLaughlin et al. 2011 [136]	Intensive trauma team training course (ITTTC): didactic lectures, case studies, and clinical simulations	Military healthcare personnel	Creates self-reported confidence	D
Meurling et al. 2013 [137]	Simulation-based team training: interactive seminars, simulation with debriefing	Intensive care	Improvement in self-efficacy. Improvement in nurse assistants’ perceived quality of collaboration and communication with physician specialists, teamwork climate, safety climate (also for nurses) and working conditions	D
Miller et al. 2012 [138]	In situ trauma simulation programme: didactic session, simulation, and debriefing	Emergency department	Improvement in teamwork and communication, this effect was not sustained after the programme was stopped	D
van der Nelson et al. 2014 [139]	Multidisciplinary simulation training with team debriefing (with emphasizes on using clinical tools)	Surgery	Improvement in safety culture, teamwork climate; deterioration in perceptions of hospital management and adequacy of staffing levels	C
Nicksa et al. 2015 [140]	Simulation of high-risk clinical scenarios followed by debriefings with real-time feedback	General surgery, vascular surgery, and cardiothoracic surgery	Improvement in communication, leadership, teamwork, and procedural ability. No significant improvement in decision making, situation awareness, and skills	C
Niell et al. 2015 [141]	Simulation-based training: didactic instruction, simulation, and debriefing	Radiology	Improvement in their ability to manage an anaphylactoid reaction, their ability to work in a team, and knowledge	B
Oseni et al. 2017 [142]	Training: video-based feedback and low-fidelity simulation	Research unit clinics and hospital (in low resource settings)	Improvement in clinical knowledge, confidence and quality of teamwork (leadership, teamwork and task management)	C
Paige et al. 2009 [143]	Repetitive training using high-fidelity simulation: Module 1 targeted teamwork competencies and Module 2 included a pre-operative briefing strategy	Operating room	Improvement in the effectiveness of promoting attitudinal change toward team-based competencies	C
Paltved et al. 2017 [144]	In situ simulation: information, simulation, and debriefing	Emergency department	Improvement in teamwork climate and safety climate	C
Pascual et al. 2011 [145]	Human patient simulation training: introduction, simulation, and video-based debriefing	Intensive care	Improvement in leadership, teamwork, and self-confidence skills in managing medical emergencies	C
Patterson et al. 2013a [146]	Multidisciplinary in situ simulations with debriefing	Paediatric emergency department	Ability to identify latent safety threats, but changes in non-technical skills	C
Patterson et al. 2013b [147]	Simulation-based training: introduction (lectures, videotapes of simulated resuscitations and case studies), simulation, and video-assisted debriefing	Paediatric emergency department	Sustained improvement in knowledge of and attitudes toward communication and teamwork behaviours	C
Pennington et al. 2018 [148]	Long distance, remote simulation training with Checklist for Early Recognition and Treatment of Acute Illness (CERTAIN)	Interdisciplinary teams in emergency situations	Improvement in global team performance: “team’s ability to complete tasks in a timely manner” and in the “team leader’s communication to the team”	C
Rao et al. 2016 [149]	Simulation team tasks: presentation, live-demonstration, and simulations	Operating room	Improvement in mean non-technical skills and concomitant increase in technical skills	D
Reynolds et al. 2011 [150]	Multidisciplinary simulation-based team training: introduction, presentation, simulation, and debriefing	Obstetrical emergencies	Improvement in knowledge, dealing with teamwork related issues, and (technical) skills (particularly relevant for obstetric nurses and for those who witness all trained obstetrical emergencies)	C
Roberts et al. 2014 [151]	Team communication, leadership and team behaviour training: didactic presentations, simulation, and debriefing	Emergency department (ad hoc emergency teams)	Changed teamwork and communication behaviour	C
Rubio-Gurung et al. 2014 [152]	In situ simulation training: briefing, simulation, and debriefing	Delivery room	Improvement in the technical skills and teamwork	B
Sandahl et al. 2013 [153]	Simulation team training: lectures, simulation, and debriefing	Intensive care	Increased awareness of the importance of effective communication for patient safety, created a need to talk, led to reflection meetings	C
Shoushtarian et al. 2014 [154]	Practical Obstetric Multi-Professional Training (PROMPT): lectures, scenario-based simulation training	Maternity	Improvement in Safety Attitude (teamwork, safety and perception of management) and clinical measures (Apgar 1, cord lactates and average length of baby’s stay in hospital)	B
Siassakos et al. 2011 [155]	Interprofessional training programme: updates on evidence-based guidelines and simple practical means of implementing them, high-fidelity simulation	Maternity unit	Positive safety culture, teamwork climate, and job satisfaction. Perceptions of high workload and insufficient staffing levels were the most prominent negative observations	D
Siassakos et al. 2011 [156]	Multiprofessional simulation training	Maternity unit	Reduction in median diagnosis–delivery interval (as indicator of teamwork)	C
Silberman et al. 2018 [157]	High-fidelity human simulation training: briefing, simulation, and debriefing	Intensive care	Facilitates teamwork, collaboration, and self-efficacy for ICU clinical practice	D
Stewart-Parker et al. 2017 [158]	Simulation-based S-TEAMS course: lectures, case studies, interactive teamwork exercises, simulated scenarios, debriefing	Operating room	Increase in confidence for speaking up in difficult situations, feeling the S-TEAMS had prevented participants from making errors, improved patient safety and team working	C
Stocker et al. 2012 [159]	Multidisciplinary in situ simulation programme (SPRinT) with debriefing	Paediatric intensive care	Impact on non-technical skills (teamwork, communication, confidence) and overall practice; less impact is perceived in technical skills	C
Sudikoff et al. 2009 [160]	High-fidelity medical simulation: didactic teaching, hands-on skills stations, case simulation, video-enhanced debriefing (with and without supplemental education)	Paediatric emergency care	Improved performance and teamwork skills; reduction in harmful actions	D
Thomas et al. 2010 [161]	Teamwork training: information session with examples and SBAR model, video clips, role playing, simulation, debriefing	Paediatric	Improvement in frequent teamwork behaviours, workload management and time to complete the resuscitation	B
Weller et al. 2016 [162]	Multidisciplinary Operating Room Simulation (MORSim) intervention: simulation, debriefing, and discussion	Operating room	Improvement in communication, culture and collaboration. But difficulties with uninterested colleagues, limited team orientation, communication hierarchies, insufficient numbers of staff exposed to MORSim and failure to prioritize time for team information sharing	D
Willaert et al. 2010 [163]	Patient-specific virtual reality (VR) simulation	Operating room	Improvement in sense of teamwork, communication, and patient safety; procedure time took longer in reality	C
Yang et al. 2017 [164]	Simulation-based interprofessional education course: preparation course, simulation, benchmarking, e-learning	Medical centre	Improvement in interprofessional collaboration attitude, self-reflection, workplace transfer and practice of the learnt skills	D
General team training				
Acai et al. 2016 [165]	Educational creative professional development workshop: various interactive team building games, activities rooted in the dramatic arts, creative printmaking session, debriefing sessions	Mental health and social care	Positive impact on teams with low team cohesion prior to the intervention. Helps staff to bond, communicate, get to know each other better and accept each other’s mistakes	D
Agarwal et al. 2008 [166]	McMaster Interprofessional Mentorship and Evaluation (MIME) programme to increase interprofessional interactions, learn more about the roles of other healthcare professionals and improve work-life satisfaction through intentional conversations at mutually agreed times	Interprofessional family health teams	No significant improvement in the QWL Survey, but participant feedback from closing workshop focus groups and evaluations was positive	C
Amaya-Anas et al. 2015 [167]	Team training: workshops, virtual modules, time-out and checklist training, and institutional actions	Operating rooms and obstetrics suites	Two or more points of improvement in the average OTAS-S scores in every phase, behaviours and sub-teams	C
Barrett et al. 2009 [168]	Intervention on lateral violence and team building: interactive groups sessions and skill-building sessions	Acute care hospital	Improvement in group cohesion and the RN-RN interaction	C
Bleakley et al. 2012 [169]	Complex education intervention: data-driven iterative education in human factors, establishing a local, reactive close call incident reporting system, and developing team self-review (briefing and debriefing)	Operating room	Improvement in teamwork climate and reduction in stress recognition. No significant improvement in job satisfaction, perception of management, working conditions, safety climate	B
Blegen et al. 2010 [170]	Multidisciplinary teamwork and communication training: presentations, videos, role playing, and facilitated discussion	Inpatient medical units	Improvement in supervisor manager expectations, organizational learning,communication openness, hospital handoffs and transitions, and non-punitive response to error	B
Brajtman et al. 2009 [171]	Interprofessional educational intervention: interactive sessions consisting of a case study, discussions and presentation	Palliative care	Improvement in leadership, cohesion, communication, coordination and conflict domains	D
Brajtman et al. 2012 [172]	Interprofessional educational intervention: self-learning module (SLM) on end-of-life delirium and interprofessional teamwork, team objective structured clinical encounter (e.g. simulation team discussion and debriefing), and a didactic “theory burst”	Long-term care facility and hospice	Improvement in knowledge and perceptions of IP competence, but does depend on the presences of the module	D
Brandler et al. 2014 [173]	Team-based learning sessions: preparation reading, tests, and application-oriented activities	Pathology	Able to solve complex problems and work through difficult scenarios in a team setting	D
Chan et al. 2010 [174]	Intervention: educational workshop (e.g. case study using role play) and structured facilitation using specially designed materials	Primary care	Improvement in patient participation, empowerment in the care process, communication and collaboration	C
Christiansen et al. 2017 [175]	Standardized Staff Development Program: educational session (i.e. lecture) and team building and resiliency session (e.g. simulation game, rounds)	Burn centre	Contributed to perceived unit cohesion and increasing satisfaction and morale	D
Chiocchio et al. 2015 [176]	Workshops integrating project management and collaboration: active, learner-centred, practice oriented strategies, feedback, and small group discussions	Interprofessional healthcare project teams	Improvement in satisfaction, perceptions of utility, self-efficacy for project-specific task work, teamwork, goal clarity, coordination, functional performance of projects	C
Cohen et al. 2016 [177]	Allied Team Training for Parkinson (ATTP): interprofessional education training on best practices and team-based care	Targeted professionals (e.g. medicine, nursing, occupational, physical and music therapies)	Improvement in self-perceived, objective knowledge, understanding role of other disciplines, attitudes toward healthcare teams, and the attitudes toward value of teams	B
Cole et al. 2017 [178]	Elective rotation of operating room management and leadership training: curriculum consisting of leadership and team training articles, crisis management text, and daily debriefings	Anaesthesiology	Improvement in teamwork, task management and situational awareness	D
Eklöf and Ahlborg 2016 [179]	Dialogue training: multiple dialogue rounds using standardized flashcards, group discussions	Hospital	Improvement in participative safety (i.e. information sharing, mutual influence and sense of having a common task) and social support from managers. Qualitative data shows a positive tendency toward trust/openness	A
Ellis and Kell 2014 [180]	Training: theory, group exercises, presentations	Paediatric ward	Improvement in team cohesiveness, effectivity, and patient care	D
Ericson-Lidman and Strandberg 2013 [181]	Intervention to constructively deal with their troubled conscience related to perceptions of deficient teamwork: assist care providers in extending their understanding of the difficult situation and find solutions to the problem through participatory action research	Elderly care	Support care providers to understand, handle and take measures against deficient teamwork. Using troubled conscience as a driving force can increase the opportunities to improve quality of care	D
Fallowfield et al. 2014 [182]	Communication skills training: workshop (e.g. presentations, exercises, discussion, role play)	Breast cancer teams	Improvement in awareness and clarity about the trial(s) discussed during the training	C
Fernandez et al. 2013 [183]	Computer-based educational intervention: computer-based training module (e.g. presentations, clinical examples, simulation-based assessment) or a placebo training module	Emergency care (and medical students)	Improvement in teamwork and patient care	B
Gibon et al. 2013 [184]	Patient-oriented communication skills training module (e.g. information, role play) and team-resource oriented communication skills training module (e.g. information, role play)	Radiotherapy	Improvement in team members’ communication skills and their self-efficacy to communicate	B
Gillespie et al. 2017 [185]	Team training programme (TEAMANATOMY): 1-h DVD (i.e. individual and shared situational awareness theory, filmed simulation pre-operative patient sign-in, and filmed simulation of time-out procedure)	Operating room	Improvement in non-technical skills (communication and interactions, situational awareness, team skills, leadership and management skills and decision making). Most significant improvement observed in surgeons. Improved use of the surgical safety checklist	C
Gillespie et al. 2017 [186]	Team training programme (TEAMANATOMY): 1-h DVD (i.e. individual and shared situational awareness theory, filmed simulation pre-operative patient sign-in, and filmed simulation of time-out procedure)	Operating room	Improvement in non-technical skills (communication and interactions, situational awareness, team skills, leadership and management skills and decision making) and the use of the surgical safety checklist. No improvement in perceived teamwork. No significant increase in perceived safety climate	C
Halverson et al. 2009 [187]	Team training: classroom curriculum, intraoperative coaching on team-related behaviours, and follow-up feedback sessions	Operating room	Improvement in perception of teamwork	C
Howe et al. 2018 [188]	Rural interdisciplinary team training programme: didactic mini-lectures, interactive case studies discussions, video presentations, role play demonstrations and the development of an action plan	Veteran affairs primary care	Improvement in teamwork	D
Kelm et al. 2018 [189]	Mindfulness meditation training using a meditation device and smartphone application at home (e.g. education, demonstration, and practice in using device, one-page summary)	Pulmonary and critical medicine physicians and ICU	Improvement in teamwork, task management, and overall performance Change in how participants responded to work-related stress, including stress in real-code situations	D
Khanna et al. 2017 [190]	Training and refresher courses on the principles of the patient-centred care medical homes: participating patient-centred medical home received coaching, learning collaborative for improving teamwork, embedded care manager	Primary care	No significant difference in perceptions of teamwork	D
Körner et al. 2017 [191]	Team coaching: identification of the expectations for team coaching (need-specific), definition of the coaching goals (task-related), development of the solution (solution-focused), maintenance of the solution (systemic)	Rehabilitation teams	Improvement in team organization, willingness to accept responsibility and knowledge integration according to staff. No significant improvement in internal participation, team leadership, and cohesion	B
Lavoie-Tremblay et al. 2017 [192]	Transforming Care at the Bedside (TCAB) programme: learning modules combined with hands-on learning	Multihospital academic health science centre	Improvement in patient satisfaction focus, overall perceived team effectiveness, perceived team skill, perceived participation and goal agreement, perceived organizational support. No significant improvement in patient experience	C
Lee et al. 2012 [193]	Communication and Patient Safety (CASP) training: practical exercises, video clips, small group discussion and other learning techniques	Emergency, outpatients, maternity, and special care nursery	Changes in behaviour at individual, team, and facility levels	C
Ling et al. 2016 [194]	BASIC (Basic Assessment and Support in Intensive Care) Patient Safety Course: blended learning course with flipped classroom approach (e.g. lectures, formative assessment, interactive sessions)	Intensive care	Improvement in teamwork within hospital units and hospital management support for patient safety, but decreased in the frequency of reporting mistakes	C
Lundén et al. 2017 [195]	Drama Workshop (warm-up activities, improvizations and Forum Theatre, reflective discussions) as a learning medium	Radiographers and registered nurses specialized in areas such as radiography, operating room and anaesthesia	Enables participants to understand each other’s priorities better and find the best way to co-operate	D
Mager et al. 2014 [196]	Team-building activities: interactive activities, discussions, case studies, readings, and/or games to promote the application of teamwork skills	Long-term and home care	Quantitatively: no statistical improvement; qualitatively: better understanding of other provider roles	C
Magrane et al. 2010 [197]	Learning in Teams model: interactive workshops, daily programme team meetings, conference calls, weekly online correspondence, and colloquium	Academic health centres	Improvement in team skills (clarifying team charge, exploring team purpose, and evaluating team process)and institutional team performance	C
Nancarrow et al. 2015 [198]	Interdisciplinary Management Tool (IMT): structured reflection through reflective exercises, facilitated sessions, evaluation conference	Community based rehabilitation or community rehabilitation servicesproviding transitional care for older people	Empowers to understand and value their own, and others’ roles and responsibilities within the team; identify barriers to effective team work, and develop and implement appropriate solutions to these	D
Prewett et al. 2013 [199]	Team training: lecture, several role plays, and guided discussion for feedback	Trauma resuscitation teams	Improvement of behavioural choices for teamwork in the trauma room. More effective responses to teamwork issues , but no affect in case of already a positive attitudes toward teamwork	D
Stephens et al. 2016 [200]	Interprofessional training course: workshops, simulated a structured debriefing technique, facilitated discussion, and sustainability strategy	Perioperative practitioners	Improvement in team behaviours (communication, coordination, cooperation and backup, leadership, situational awareness); recognizing different perspectives and expectations within the team; briefing and debriefing	D
Webb et al. 2010 [201]	Emotional intelligence coaching: homework assignments, coaching sessions, goal setting	Family medicine	Decline in teamwork rating and no improvement on competences	D
Tools: Structuring teamwork: SBAR				
Beckett et al. 2009 [202]	SBAR Collaborative Communication Education (e.g. didactic content, role play, and an original DVD demonstrating traditional and SBAR communication)	Hospital paediatrics/perinatal services department	Improvement in communication, collaboration, satisfaction, and patient safety outcomes	C
Clark et al. 2009 [203]	PACT (Patient assessment, Assertivecommunication, Continuum of care, Teamwork with trust) Project, aimed at improving communication between hospital staff at handover: 2 communication tools based on SBAR: Handover prompt card and reporting template	Private hospital	improvement in communication, handover, and confidence in communicating with doctors	C
Costa and Lusk 2017 [204]	SBAR educational session	Behaviour health clinicians in correctional facilities	Marginal improvement in communication and team structure	D
Donahue et al. 2011 [205]	EMPOWER project: an interdisciplinary leadership-driven communication programme (Educating and Mentoring Paraprofessionals On Ways to Enhance Reporting) using SBAR	Hospital	Improvement in communication from paraprofessional staff to professional staff, no significant changes in rapid events reports	C
Martin et al. 2015 [206]	Huddles structured with SBAR with an educational session	Paediatric emergency department	Improvement in teamwork, communication, and nursing satisfaction	C
Randmaa et al. 2014 [207]	SBAR and implementation strategies (e.g. modified SBAR card, in-house training course, information material and observation)	Anaesthetic clinics	Improvement in between-group communication accuracy, safety climate, the proportion of incident reports due to communication errors	C
Renz et al. 2013 [208]	SBAR protocol and training	Nursing homes	Mixed results regarding the nurse satisfaction with nurse-medical provider communication	D
Rice et al. 2010 [209]	Interprofessional intervention: semi-scripted four-step process during all patient-related interactions (i.e. name, role, issue, and feedback)	General internal medicine	No changes in communication and collaboration between health professionals	D
Sculli et al. 2015 [210]	Effective Followership Algorithm: 3Ws (what I see; what I’m concerned about; what I want), 4-Step Assertive Tool, Engage team, Chain of command	Paediatric and adult operating rooms	Improvement in safety culture, teamwork, team performance	C
Ting et al. 2017 [211]	SBAR Collaborative Communication Education: educational session, case-based discussion, video demonstration on traditional and SBAR communication	Obstetrics department	Improvement in teamwork climate, safety climate, job satisfaction, and working conditions	D
Weller et al. 2014 [212]	Video-intervention teaching SNAPPI tool: Stop the team; Notify of the patient’s status; Assessment of the situation; Plan what to do; Priorities for actions; and Invite ideas	Anaesthesiology	Improvement in SNAPPI score, number of diagnostic options, information sharing. No significant improvement in information probe sharing and medical management (in intervention group)	C
Tools: Structuring teamwork: (De)briefing checklist				
Berenholtz et al. 2009 [244]	Standardized one-page briefing and debriefing tool	Operating room	Improvement in interdisciplinary communication and teamwork	C
Bliss et al. 2012 [213]	Comprehensive surgical safety checklist (using pre-operative briefing and post-operative debriefing checklists) and a structured team training curriculum	Surgery	Decrease in 30-day morbidity. Cases with safety-compromising events (e.g. inadequate communication, decision making), had higher rates of 30-day morbidity	B
Böhmer et al. 2012 [214]	Modified perioperative surgical safety checklist	Operating room	Improvement in interprofessional coordination and communication	D
Böhmer et al. 2013 [215]	Perioperative safety checklists	Anaesthesiology and traumatology	Improvement in verification of written consent for surgery, clear marking of the surgical site, time management, better informed about the patients, the planned operation, and the assignment of tasks during surgery in both short and long terms. Decrease in communication over longer time periods.	B
Boet et al. 2011 [245]	Self-debriefing versus instructor debriefing	Hospital	Improvement in situational awareness, teamwork, decision making, task management, total non-technical skills, regardless of the type of debriefing received	B
Boet et al. 2013 [246]	Interprofessional within-team debriefing compared to an instructor-led debriefing	Operating room	Improvement in team performance regardless of the type of debriefing. No significant difference in the degree of improvement between within-team debriefing and instructor-led debriefing	C
Cabral et al. 2016 [216]	Standardized, comprehensive time-out and a briefing/debriefing process using surgical safety checklist	Surgery	Improvement of nurses’ perception of communication. No significant improvement of surgeons and technologists perception of communication	C
Calland et al. 2011 [220]	Surgical safety checklists (intervention group included a basic team training using a pre-procedural checklist)	Surgery	Improvement in team behaviour, defined as discrete, objective, observable shared communication behaviours; more likely to involve positive safety-related team behaviours such as case presentations, explicit discussions of roles and responsibilities, contingency planning, equipment checks, and post case debriefings; no significant differences in situational awareness	A
Dabholkar et al. 2018 [218]	Customized surgical safety checklist	Surgery	Improvement in verification of patient’s identity, awareness of operating team members’ names and roles, practice of displaying radiological investigation during surgery, pre-check of equipment and communication	B
Dubois et al. 2017 [219]	Person-centred endoscopy safety checklist (introduces during seminars and training)	Endoscopy unit	Improvement in quality of collaboration with nurses and perception. No differences in teamwork	D
Einav et al. 2010 [247]	Pre-operative team briefings (briefing protocol and poster)	Operating room	25% reduction in the number of non-routine events when briefing was conducted and a significant increase in the number of surgeries in which no non-routine event was observed. Team members evaluated the briefing as most valuable for their own work, the teamwork, and patient safety	C
Erestam et al. 2017 [220]	Revised surgical safety checklist	Operating room	No significant change in teamwork climate. Lack of adherence to the checklist was detected	C
Everett et al. 2017 [221]	Critical event checklists	Surgical daytime facility	No improvement in medical management or teamwork (during simulation)	C
Gleicher et al. 2017 [248]	Standardized handover protocol consisting of a handover content checklist and a “sterile cockpit” time-out	Cardiovascular intensive care	Improvement in teamwork, content received and patient care planning	C
Gordon et al. 2014 [222]	Pre-procedure checklist	Cardiac catheterization laboratory	No improvement in complication rates, overall team and safety attitudes	C
Hardy et al. 2018 [223]	Malignant hyperthermia checklist	Anaesthesiology	Improvement in non-technical skills in the experiment group. Higher self-reported stress in the experiment group	C
Haugen et al. 2013 [224]	Surgical safety checklist	Operating room	Improvement in frequency of events reported and adequate staffing. No significant improvement in patient safety, teamwork within units, communication on error, hospital management promoting safety	B
Haynes et al. 2011 [225]	Checklist-based surgical safety intervention	Operating rooms	Improvement in teamwork and safety climate	C
Helmiö et al. 2011 [226]	Surgical safety checklist	Operating room	Improvement in verification of the patient’s identity, awareness of the patient’s medical history, medication and allergies, knowledge of the names and roles among the team members, discussion about possible critical events, recording post-operative instructions, communication between team members	B
Howe et al. 2014 [249]	Long-term care team talk programme involved regularly scheduled 5-min debriefing sessions at the end of the day shift led by a rotating schedule of certified nurse	Transitional care unit in long-term care facility	Improvement in co-worker and supervisor support, teamwork and communication, job demands and decision authority, characteristics of the unit and intent to leave/transfer unit	C
Jing and Honey 2016 [227]	Robotic-assisted laparoscopic radical prostatectomy checklist	Operating room	Improvement in teamwork, time efficiency, higher confidence levels and more comprehensive operating room setup	D
Kawano et al. 2014 [228]	Surgical safety checklist	Surgery	Improvement in the Safety Attitude Scores	C
Kearns et al. 2011 [229]	Modified surgical safety checklist	Obstetric theatre	Improvement in interprofessional communication, familiarity with team members, and checklist compliance	C
Kherad et al. 2018 [230]	Endoscopy checklist implementation (with lectures by quality officers)	Endoscopy	Improvement in team work and communication, patient perception of team communication and teamwork. No significant improvement in team perception	C
Khoshbin et al. 2009 [250]	“07:35 huddles” (pre-operative OR briefing following 4 elements) and “surgical time-outs” (pre-operative OR briefing following 9 elements)	Paediatric hospital	Especially for the nursing personnel, change the notion of individual advocacy to one of teamwork and being proactive about patient safety	C
Lepanluoma et al. 2014 [231]	Surgical safety checklist	Operating room	Improvement in communication between the surgeon and the anaesthesiologist. Safety-related issues were better covered. No improvement in awareness. Improvement in unplanned admission rates and number of wound complications	D
Lingard et al. 2008 [251]	Team briefing structured by a checklist	General surgery	Improvement in number of communication failures and proactive and collaborative team communication	C
Low et al. 2013 [232]	“Flow checklists” at high-risk points in the patient surgical journey, in addition to the surgical safety checklist	Ambulatory surgery centre	Improvement in the perception of patient safety	D
McLaughlin et al. 2014 [252]	Time-Out Process: (1) team member introductions, (2) safety statement by the time-out leader, (3) addition of two supplemental items to the institutional checklist, and (4) pre-incision Surgical Care Improvement Project measures	Neurosurgery in operating room	Improvement in the perception of patient safety, team spirit, voice safety concerns. Does not necessarily reinforce teamwork.	D
Merrell et al. 2018 [233]	Emergency manual consisting of a set of crisis checklists or cognitive aids	Operating room	Enabled perceived effective team functioning through reducing stress, fostering a calm working environment and improvement teamwork and communication	D
Mohammed et al. 2013 [234]	Obstetric safe surgery checklist	Anaesthetists and obstetricians	Improvement in communication of caesarean section grade (urgency) between obstetricians and anaesthetists	C
Molina et al. 2016 [235]	Surgical safety checklists	Operating room	Improvement in respect, clinical leadership, assertiveness, coordination, and communication	A
Nadler et al. 2011 [253]	Debriefings using video recordings	Neonatal resuscitation	Improvement in teamwork	C
Nilsson et al. 2010 [236]	Pre-operative checklist during time-out	Operating room	Improvement in “team feeling”	D
Norton et al. 2016 [237]	Novel paediatric surgical safety checklist	Operating room at paediatric hospital	Reduced complications and errors and improved patient safety, communication among team members, teamwork in complex procedures, efficiency in the operating room, prevented or averted an error or a complication	C
Nundy et al. 2008 [254]	Pre-operative briefings using a standardized format (with training session)	Operating room	Reduction in unexpected delays and communication breakdowns leading to delays	B
Paige et al. 2009 [255]	Pre-operative briefing protocol	Operating room	Improvement in pre-operative briefing and overall team interaction; no significant improvement in procedure time	D
Pannick et al. 2017 [256]	Prospective clinical team surveillance (PCTS): structured daily interdisciplinary briefings to capture staff concerns, with organizational facilitation and feedback	Medical ward	Improvement in safety and teamwork climates, reduction in excess length of stay (eLOS)	B
Papaconstantinou et al. 2013 [238]	Surgical safety checklist	Surgery	Improvement in the awareness of patient safety and quality of care, the perception of the value of and participation in the time-out process, surgical team communication, and in the establishment and clarity of patient care needs	B
Papaspyros et al. 2010 [257]	Pre-operative briefing with checklist and debriefing	Cardiac operating room	Improvement in communication	D
Sewell et al. 2011 [239]	Educational programme focused on using the surgical safety checklist	Orthopaedic surgery	Increase in checklist use, believe that the checklist improved team communication; checklist use was not associated with a significant reduction in early complications and mortality in patients undergoing orthopaedic surgery	B
Skåre et al. 2018 [258]	Video-assisted, performance-focused debriefings	Delivery	Improvement in Neonatal Resuscitation Performance Evaluation (NRPE) score: group function/communication, preparation and initial steps and positive pressure ventilation	C
Steinemann et al. 2016 [259]	Structured physician-led briefing (using a checklist)	Trauma care	Improvement in T-NONTECH leadership scale (not the other domains) and task completions (not for all scenarios)	C
Takala et al. 2011 [240]	Surgical safety checklist	Operating room	Improvement in confirming patient’s identity, knowledge of names and roles among team members, discussing critical events, and fewer communication failures	A
Tscholl et al. 2015 [241]	Anaesthesia pre-induction checklist, in addition to the surgical safety checklist	Anaesthesiology	Improvement in information exchange, knowledge of critical information, perception of safety in anaesthesia teams, perceived teamwork	A
Urbach et al. 2014 [242]	Surgical safety checklist	Operating room	Implementation is not associated with significant reductions in operative mortality or complications	B
Wagner et al. 2014 [260]	Mental health huddles (similar to safety briefings) to support staff in discussing and managing client responsive behaviours	Long-term care	improvement in staff collaboration, teamwork, support, and communication	D
Weiss et al. 2017 [261]	After events reviews (AER): assertiveness-specific AER (ASAER) versus teamwork-generic AER (TGAER)	Healthcare teams	Improvement in nurses speaking up following the ASAER in comparison to TGAER and higher levels of hierarchy-attenuating beliefs following the ASAER in comparison to TGAER	C
White et al. 2017 [243]	Four-day pilot course for implementation of surgical safety checklist	Hospital (low-income setting)	Improvement in learning, behaviour and organizational change (not hierarchical culture)	D
Whyte et al. 2009 [262]	Structured pre-operative team briefings (using a checklist)	Pre-operative teams	Five types of negative events: the briefings could mask knowledge gaps, disrupt positive communication, reinforce professional divisions, create tension, and perpetuate a problematic culture	D
Zausig et al. 2009 [263]	Two different training groups: one included extensive debriefing of NTS (resource management, planning, leadership and communication) and medical management and the other included a simpler debriefing that focused solely on medical management	Anaesthesiology	Improvement in non-technical skills; no differences between the groups	D
Tools: Structuring teamwork: Rounds				
Genet et al. 2014 [264]	Respiratory therapist (RT)-led interdisciplinary rounds using a scripted tool (with education session)	Neonatal ICU	Improvement in communication, teamwork, and timeliness of completing respiratory orders	B
Henkin et al. 2016 [265]	Bedside rounding: inclusion of nurses in morning rounds with the medicine teams at the patients’ bedside, using a checklist	General medicine inpatient teaching unit	Improvement in the perceptions of nurse–physician teamwork	C
Li et al. 2018 [266]	Interprofessional Teamwork Innovation Model (ITIM): structured daily rounds	Academic medical centre	Improvement in communication among team members and overall time savings. Reduction in 30-day same-hospital readmissions, no impact on 30-day same-hospital ED visits or costs	B
O’Leary et al. 2010 [267]	Structured Interdisciplinary Rounds combined a structured format for communication and a forum for regular interdisciplinary meetings	Tertiary care teaching hospital	Improvement in teamwork climate in intervention group (compared to control group)	B
O’Leary et al. 2011 [268]	Structured Interdisciplinary Rounds: combined a structured format for communication with a forum for regular interdisciplinary meetings	General medical unit in hospital	Improvement in quality of communication and collaboration with hospitalists, teamwork and safety climate	C
O’Leary et al. 2015 [269]	Structured Interdisciplinary Rounds and prepared nurse–physician co-leadership	General medical units	Improvement in teamwork but no reduction in Adverse Events	C
Young et al. 2017 [270]	Multidisciplinary Bedside Rounding Initiative, which included creating nursing availability, streamlining provider communication, and performance monitoring and feedback	Hospital	Improvement in teamwork climate, nurse job satisfaction, and early discharges	D
Tools: Facilitating teamwork				
Butler et al. 2018 [271]	Telemedicine technology in care delivery	Emergency care	No differences in teamwork between control and experiment groups. Higher workload in experiment group	B
Chu-Weininger et al. 2010 [272]	Remote monitoring by intensivists using telemedicine technology (tele-ICU)	Intensive care	Improvement in teamwork climate and safety climate	B
Doyle et al. 2016 [273]	Remote information technology (education session, teleconferences, web-based team case presentations)	Mental health services for older people	Improvement in professional development, perceived peer support, team building, cohesion, and reduce travel time	D
Foo et al. 2015 [274]	Mobile task management tool (digitize patient flow and provide real-time visibility over clinical decision making and task performance)	Acute general surgical service	Improvement in working efficiency of junior clinical staff	C
Letchworth et al. 2017 [275]	MedNav; a decision support tool on a tablet or mobile phone with integrated vocal prompts and visual cues	Maternity teams	Improvement in teamwork based on all domains of Clinical Teamwork Scale and Global Assessment of Obstetric Team Performance	B
O’Connor et al. 2009 [276]	Using wireless e-mail in order to send information-rich, specific, legible, and time-stamped messages	Intensive care	Improvement in communication, team relationships, staff satisfaction, and patient care	D
Yeh et al. 2016 [277]	Ping-pong-type multidisciplinary reflective e-communication (within web-based integrated information platform)	Radiation oncology	Higher Timeliness, Notating convenience, Information completeness, Feedback convenience, Communication confidence, Communication effectiveness, Review convenience and overall satisfaction	C
Tools: Triggering teamwork				
Aberdeen and Byrne 2018 [278]	Concept mapping visually representing a patient’s situation	Residential aged care facilities	Improvement in effectiveness of care planning and knowledge increase of dementia care	D
Ainsworth et al. 2013 [279]	Door Communication Card (DCC) to improve goal alignment	Surgical ICU academic military medical	No improvement in goal alignment	D
Bennett et al. 2015 [280]	Sharing clinical cases and stories about patients (during workshops)	Primary care clinical setting	Helped in bonding around their shared mission of patient-centred care, build supportive relationships, enhance compassion for patients, communicate and resolve conflict, better understand workflows and job roles, develop trust, and increase morale	D
Daley et al. 2012 [281]	Clinical dashboard system	Acute elderly care	Improvement in access to information, communication and information sharing, staff awareness, and data quality	D
O’Neil et al. 2017 [282]	Thought for the Day (TOD) intervention; a short reflection on a piece of poetry, music, or religious writing	Inpatient palliative care	Improvement in perception of teamwork. Coming together as an interdisciplinary team for a time to reflect is valued	D
Siegele 2009 [283]	The Daily Goals Tool (DGT) and Daily Goals Tool Reference (DGTR)	Surgical intensive care	Helps in simplifying complex tasks, improving teamwork, promoting effective communication and shared decision making, and enhancing patient safety	D
Stoller et al. 2010 [284]	Respiratory therapy (RT) business scorecard that compared target goals with actual monthly performance	Respiratory therapy departments	Improvement in teamwork among RT departments and outcomes	D
Organizational (re)design				
Barry et al. 2016 [285]	Behavioural Health Interdisciplinary Program (BHIP) team model as an innovative approach to transform VHA general outpatient mental health delivery, include holding daily huddles and longer weekly interdisciplinary team meetings	Veterans Health Administration mental health care	Improvement in teamwork and patient care and has potential to improve staff working relationships, communication, collaboration, morale, and veteran treatment consistency	D
de Beijer et al. 2016 [286]	Clinical pathways: standardizing treatment and communication methods, delegating tasks from medical specialists to nurses, and providing nurses with their own consultation room	Orthopaedic hand unit outpatient clinic	Improvement in the actual communication and collaborative problem-solving skills concerning standard patients	D
Clements et al. 2015 [287]	Allocating the most senior nurse as team leader of trauma patient assessment and resuscitation	Emergency department	Improvement in understanding of their role, “intimidating personality”, and nursing leadership	C
Deneckere et al. 2013 [288]	Care pathways: (1) Formative evaluation of the teams’ performance before implementation, (2) Evidence-based KI, and (3) Training in pathway development	Acute hospital	Improvement in conflict management, team climate for innovation, level of organized care, risk of burnout, emotional exhaustion, and competence. No significant improvement in relational coordination	B
Fernandez et al. 2010 [289]	Two models: The multifaceted Shared Care in Nursing (SCN) model of nursing careinvolved team work, leadership and professional development. In the Patient Allocation (PA) model one nurse was responsible for the care of a discrete group of patients	General medical and surgical wards in tertiary teaching hospital	The two models of care support most aspects of interdisciplinary and intra-disciplinary communication	C
Fogel et al. 2016 [290]	Patient-focused primary care redesign	Continuity clinic settings	Improvement in teamwork training, teamwork among residents, perception of overall quality of care in clinic, and that physicians, nurses, and administrative staff worked together to optimize patient flow	C
Frykman et al. 2014 [291]	Multiprofessional teamwork involving changes in work processes, with task-generated feedback, managerial feedback, aimed at increasing interprofessional collaboration	Emergency department	Enabled teamwork	C
Greene et al. 2015 [292]	Innovative compensation model: replaced fee-for-service payment with a largely team based, quality-focused payment, 40% of compensation was based upon the clinic-level quality performance, and an additional 10% was based upon the clinic-level patient’s experience	Primary care	Mixed results: quality improvement for the team and less patient “dumping,” or shifting patients with poor outcomes to other clinicians, but also lack of control and colleagues riding the coattails of higher performers. mixed results: greater interaction with colleagues, but also an increase in tension	C
Hern et al. 2009 [293]	Quality improvement intervention: creation of team structures linking faculty advisors and residents with patients, intra-team management of office tasks, and the implementation of multidisciplinary team meetings	Family medicine	Improvement in perceptions of continuity of patient care, office efficiency, and team communication	C
Hung et al. 2018 [294]	Redesign consisting of multiple workflow changes: (1) “5S” standardization of medical equipment, supplies and education materials in patient exam rooms, (2) redesign of call centre functions, (3) co-location of existing care teams and (4) redesign of care team roles and workflows	Ambulatory care primary care departments	Improvement in teamwork, participation in decisions to improve care by physicians, engagement among physicians and motivation among Non-physicians staff	C
O’Leary et al. 2009 [295]	Localizing physicians to specific patient care units	Hospital	Nurses and physicians wereable to identify one another and communicated more frequently	B
Pan et al. 2017 [296]	An operating room (OR) assistant using an instructional supervision programme	Operating room	Improvement in first cases that started on time, percentage of teamwork score and patient satisfaction	B
Parush et al. 2017 [297]	Employ technological cognitive aids at ED	Emergency Department	Improvement in teamwork; overall communication, situational awareness (as measured by CTS and not SAGAT), and decision making	D
Pati et al. 2015 [298]	Decentralized unit operations and the corresponding physical design	Inpatient units	Potentially improvement in quality of work	D
Stavroulis et al. 2013 [299]	Integrated theatre environment: a superior operating environment in which the laparoscopic equipment and multiple flat-screen monitors are permanently installed to be operational on demand inside the theatre	Operating room	Improvement in perceived efficiency, teamwork and stress levels	C
Stepaniak et al. 2012 [300]	Fixed operating room (OR) teams for a day instead of OR teams that vary during the day	Operating room (bariatric surgery)	Reduced procedure durations and improved teamwork and safety climate, without adverse effects on patient outcomes	B
Programme				
Basson et al. 2018 [301]	Multifaceted intervention consisting of monthly walking rounds by the director and an interactive learning session focused of feedback of culture data, educational training programme, and unit-based programme for safety	Veterans administration hospital leaders	No improvement on most items of the SAQ and AHRQ Hospital Safety Survey. Improvement in responding to errors and expressing disagreement with physicians. Decrease of perception of leadership’s safety efforts and levels of staffing	D
Bunnell et al. 2013 [302]	For each identified risk area, agreements about roles, responsibilities and behaviours of each team member were made. Tools were developed and systems modified to enhance situational awareness and a shared mental model among team members, and to support implementation of the agreements	Ambulatory clinical oncology practice	Improvement in patient satisfaction scores regarding coordination of care, efficiency safety of care, more respectful behaviour, relationships among team members. No significant improvement in non-communication	C
Braithwaite et al. 2012 [303]	System-wide intervention promoting interprofessional collaboration; implementing educational workshops and seminars, feedback sessions, project, and other initiatives	Health professionals across entire health system	Most agreement on improvement in sharing of knowledge between professions and improved quality of patient care, and least agreement that between-professional rivalries had lessened and communication and trust between professions improved	B
Carney et al. 2011 [304]	Medical team training programme: preparations, learning sessions, implementing projects including briefing and debriefing, coaching	Operating room in Veterans Health Administration	Improved perceptions of safety climate	B
Carney et al. 2011 [305]	Medical team training programme: preparations, learning sessions, implementing projects including briefing and debriefing, coaching	Veterans Health Administration	Improvement in teamwork climate	B
Costello et al. 2011 [306]	OR Transformation Project: OR day redesign, workflow, human resources analysis, supply and technology, and quality of work life	Operating room	Improvement in work practices, recognition/ compensation, communication, commitment, physical/environmental safety, teamwork, and respect	C
Ginsburg and Bain 2017 [307]	Multifaceted intervention programme to promote speaking up and teamwork consisting a role-playing simulation workshop, discussion briefings and other department-led initiatives such as 10-min staff huddles	Emergency department and intensive care	Improvement in team climate score at follow-up	B
Hilts et al. 2013 [308]	The Quality in Family Practice (QIFP) programme encompasses clinical and practice management using a comprehensive tool of family practice indicators	Academic primary care clinics	Improvement in understanding of team roles and relationships, teamwork, flattening of hierarchy through empowerment	D
Hsu et al. 2015 [309]	Multifaceted intervention included Comprehensive Unit-based Safety Program (CUSP), the daily goals communication tool, and 5 evidence-based practices (i.e. hand washing, using full-barrier precautions during the insertion of central venous catheters, cleaning the skin with chlorhexidine, avoiding the femoral site, and removing unnecessary catheters)	Adult intensive care	Improvement in safety climate, job satisfaction, and working conditions	B
Hsu et al. 2014 [310]	Team Resource Management (TRM) programme: simulative learning workshop (e.g. lectures, videos, case-based interactive discussions), focus group interviews, develop TRM-based checklists, working sheets, and re-designed organ procurement and transplantation processes, video skill demonstration and training, case reviews and feedback activities	Hospital	No significant improvement on teamwork (i.e. teamwork framework, leadership, situational awareness, communication, mutual support); no error in communication or patient identification was noted	C
Je et al. 2013 [311]	Hospital-wide quality improvement programme: forming committee to review the system, implemented a dedicated communication system, standardizationon of role, training, implementing a standard reporting system	Hospital	Improvement in safety attitude (i.e. sharing information, training, medical error reporting, safety climate, job satisfaction, communication, hospital management quality)	B
Kotecha et al. 2015 [312]	Quality Improvement Learning Collaborative Program: learning sessions, action periods to develop improvement plans, and summative congresses supported by QI coaches, teleconferences, and a web-based virtual office	Primary care	Improvement in trust and respect for each other’s clinical, administrative roles, collegial relationships, collapse professional silos, communication, and interdisciplinary collaboration	D
Lin et al. 2018 [313]	Safety Program for Surgery: Comprehensive Unit-based Safety Program (CUSP) and individualized bundles of interventions	Hospitals	Improvement in overall perception/patient safety, teamwork across units, management support for patient safety, non-punitive response to error, communication openness, frequency of events reported, feedback/communication about error, organizational learning/continuous Improvement, supervisor/manager expectations and actions promoting safety, and teamwork within units	B
McArdle et al. 2018 [314]	Safety Program for Perinatal Care (SPPC, adapted CUSP): TeamSTEPPS teamwork and communication framework and tools, applying safety science principles (standardization, independent checks, and learn from defects), and establishing an in situ simulation programme	Labour and delivery	Improvement in the se of shoulder dystocia safety strategies, in situ simulation, teamwork and communication, standardization, learning from defects, and independent checks	B
McCulloch et al. 2017 [315]	Four-month safety improvement interventions, using teamwork training (TT), systems redesign and standardization (SOP), Lean qualityimprovement, SOP + TT combination, or Lean+TT combination	Operating room	TT: improvement in non-technical skills and WHO compliance, but not technical performance. Systems interventions (Lean and SOP): improvement in non-technical skills and technical performance, WHO compliance. Combined interventions: improvement in all performance measures except WHO time-out attempts, whereas single approaches improved WHO compliance less and failed to improve technical performance	B
Neily et al. 2010 [316]	Medical team training programme: preparation, learning session, implementing briefings, debriefings and other projects (i.e. SBAR, Interdisciplinary rounds, Fatigue management), follow-up coaching	Surgical care in Veterans Health Administration	Improvement in teamwork, efficiency, avoiding an undesirable event	C
Neily et al. 2010 [5]	Medical team training programme: preparation, learning session, implementing projects, follow-up coaching	Operating room in Veterans Health Administration	Lower surgical mortality and improvement in open communication and staff awareness	A
Pettker et al. 2011 [317]	Comprehensive Obstetrics Patient Safety Program: (1) obstetrics patient safety nurse, (2) protocol-based standardization of practice, (3) CRM training, (4) oversight by a patient safety committee, (5) 24-h obstetrics hospitalist, and (6) anonymous event reporting system	Hospital	Improvement in proportion of staff members with favourable perceptions of teamwork culture, safety culture, job satisfaction, and management. No significant improvement in stress recognition	B
Pitts et al. 2017 [318]	Comprehensive Unit-based Safety Program (CUSP): training, safety assessment, select safety priorities	Primary care	No significant improvement in safety climate and teamwork	D
Pronovost et al. 2008 [319]	Comprehensive Unit-based Safety Program including implementing CUSP (i.e. 6-step iterative process), daily goals communication strategy, and toolkit included materials for staff education, redesign of work processes, support of local opinion leaders, and evaluation of performance	Intensive care	Improvement in teamwork climate	B
Sexton et al. 2011 [320]	Comprehensive Unit-based Safety Program (CUSP): educate teams, identify, prioritize, and eliminate patient safety hazards, senior leader’s role, tools for learning and improving communication	Intensive care	Improvement in safety climate	B
Stapley et al. 2017 [321]	The Situation Awareness For Everyone (SAFE) programme: huddle, SBAR, and paediatric early warning systems (PEWS)	Clinical wards	Improvement in awareness of important issues, communication, teamwork, and a culture of increased efficiency, anticipation and planning on the ward. But added pressure on staff time and workload, and the potential for junior nurses to be excluded from involvement	D
Timmel et al. 2010 [322]	Comprehensive Unit-Based Safety Program (CUSP) including 6 steps: Science of safety training educational curriculum, Identify safety hazards, Senior executive partnership, Learn from defects, Implement improvement tools, such as team-based goals sheet, including nurses on rounds to form an interdisciplinary team	Surgical inpatient units	Improvement in safety climate, teamwork climate, and nurse turnover rates	B
Wolf et al. 2010 [323]	Medical team training programme: preparation, classroom learning session, checklist-guided briefings and debriefings, formation of a problem-solving Executive Committee, follow-up and feedback	Operating room in Veterans Health Administration	Improvement in case delays, mean case score, frequency of pre-operative delays, handoff issues, equipment issues/delays, perceived management and working conditions. No significant improvement in teamwork climate, safety climate, job satisfaction, stress recognition	B

Studies can also be upgraded or downgraded based on additional criteria. For example, a study is downgraded by one category in the event there are important inconsistencies. Detailed information is provided as additional material (see Additional file 2).

### Organization of results

The categorization of our final set of 297 articles is the result of three iterations. First, 50 summarized articles were categorized using the initial categorization: team training (subcategories: CRM-based training, simulation training, interprofessional training, and team training), tools, and organizational intervention [8]. Based on this first iteration, the main three categories (i.e. training, tools, and organizational interventions) remained unchanged but the subcategorization was further developed. Training, related to the subcategory “CRM-based training”, “TeamSTEPPS” was added as a subcategory. The other subcategories (i.e. simulation training, interprofessional training, and team training) remained the same. Tools, the first draft of subcategories, entailed Situation, Background, Assessment, and Recommendation (SBAR), checklists, (de)briefing, and task tools. Two subcategories of organizational intervention (i.e. programme and (re)design) were created, which was also in line with the content of this category in the original literature review. Second, 50 additional articles were categorized to test and refine the subcategories. Based on this second iteration, the subcategories were clustered, restructured and renamed, but the initial three main categorizations remained unaffected. The five subcategories of training were clustered into principle-based training, method-based training, and general team training. The tools subcategories were clustered into structuring, facilitating, and triggering tools, which also required two new subcategories: rounds and technology. Third, the remaining 197 articles were categorized to test the refined categorization. In addition, the latter categorization was peer reviewed. The third iteration resulted in three alterations. First, we created two main categories based on the two subcategories “organizational (re)design” and “programme” (of the third main categorization). Consequently, we rephrased “programme-based training” into “principle-based training”. Second, the subcategories “educational intervention” and “general team training” were merged into “general team training”. Consequently, we rephrased “simulation training” into “simulation-based training”. Third, we repositioned the subcategories “(de)briefing” and “rounds” as structuring tools instead of facilitating tools. Consequently, we merged the subcategories “(de)briefing” and “checklists” into “(de)briefing checklists”. Thereby, the subcategory “technology” became redundant.

## Results

Four main categories are distinguished: training, tools, organizational (re)design, and programme. The first category, *training*, is divided in training that is based on specific principles and a combination of methods (i.e. CRM and Team Strategies and Tools to Enhance Performance and Patient Safety (TeamSTEPPS)), a specific training method (i.e. training with simulation as a core element), or general team training, which refers to broad team training in which a clear underlying principle or specific method is not specified. The second category, *tools*, are instruments that are introduced to improve teamwork by structuring (i.e. SBAR (Situation, Background, Assessment, and Recommendation), (de)briefing checklists, and rounds), facilitating (through communication technology), or triggering (through monitoring and feedback) team interaction. Structuring tools partly standardize the process of team interaction. Facilitating tools provide better opportunities for team interaction. Triggering tools provide information to incentivize team interaction. The third category, *organizational (re)design*, refers to (re)designing structures (through implementing pathways, redesigning schedules, introducing or redesigning roles and responsibilities) that will lead to improved team processes and functioning. The fourth category, a *programme*, refers to a combination of the previous types of interventions (i.e. training, tools, and/or redesign). Table 2 presents the (sub)categorization, number of studies, and a short description of each (sub)category.

**Table 2:** Categorization of results

Interventions	*n*	Description
1. Training	174	“A systematic process through which a team is trained to master and improve different aspects of team functioning.” [8]
1.1 Principle-based training		
a. CRM-based training	40	“Training based on a management concept used in the aviation industry to improve teamwork. CRM encompasses a wide range of knowledge, skills, and attitudes including communication, situational awareness, problem solving, decision making, and teamwork.” [8]
b. TeamSTEPPS	28	A specific set of strategies and techniques, aimed at optimizing patient outcomes by improving communication and teamwork skills among healthcare professionals. (https://www.ahrq.gov/teamstepps/index.html)
1.2 Method-based training: Simulation-based training	69	“Training that recreates characteristics of the real world.” [8]
1.3 General team training	37	General team training includes studies that each has a unique combination of principles and learning methods.
2. Tools	83	Specific instruments that teams can use to improve teamwork [8]
2.1 Structuring tools		Tools that are used to partly standardize the process of team interaction.
a. SBAR	11	The SBAR (Situation, Background, Assessment, Recommendation) is a framework for communication between team members about a patient’s condition. (www.ihi.org)
b. (De)briefing checklist	51	A tool that creates an opportunity for professionals to systematically communicate and discuss (potential) issues before or after delivering care to a patient, based on a structured format of elements/topics; checklist.
c. Rounds	7	A structured interdisciplinary meeting around a patient.
2.2 Facilitating tools	7	Tools (often technology) that facilitate communication between team members.
2.3 Triggering tools	7	Tools that help provide information (e.g. dashboards) to incentivize team interaction.
3. Organizational (re)design	16	Design or redesign of organizational structures with the aim of improving team processes and team functioning.
4. Programme	24	A combination of interventions (training, tools, and/or organizational (re)design) bundled in a program that aims to improve team functioning.
Total	297	

### Overall findings

#### Type of intervention

The majority of studies evaluated a training. Simulation-based training is the most frequently researched type of team training.

#### Setting

Most of the articles researched an acute hospital setting. Examples of acute hospital settings are the emergency department, operating theatre, intensive care, acute elderly care, and surgical unit. Less attention was paid to primary care settings, nursing homes, elderly care, or long-term care in general.

#### Outcome

Interventions focused especially on improving non-technical skills, which refer to cognitive and social skills such as team working, communication, situational awareness, leadership, decision making, and task management [21]. Most studies relied on subjective measures to indicate an improvement in team functioning, with only a few studies (also) using objective measures. The Safety Attitude Questionnaire (SAQ) and the Non-Technical Skills (NOTECHS) tool are frequently used instruments to measure perceived team functioning.

#### Quality of evidence

A bulk of the studies had a low level of evidence. A pre- and post-study is a frequently used design. In recent years, an increasing number of studies have used an action research approach, which often creates more insight into the processes of implementing and tailoring an intervention than the more frequently used designs (e.g. Random Control Trial and pre-post surveys). However, these valuable insights are not fully appreciated within the GRADE scale.

The findings per category will be discussed in greater detail in the following paragraphs.

### Training

CRM and TeamSTEPPS are well-known *principle-based trainings* that aim to improve teamwork and patient safety in a hospital setting. Both types of training are based on similar principles. *CRM* is often referred to as a training intervention that mainly covers non-technical skills such as situational awareness, decision making, teamwork, leadership, coping with stress, and managing fatigue. A typical CRM training consists of a combination of information-based methods (e.g. lectures), demonstration-based methods (e.g. videos), and practice-based methods (e.g. simulation, role playing) [9]. However, CRM has a management concept at its core that aims to maximize the use of all available resources (i.e. equipment, time, procedures, and people) [324]. CRM aims to prevent and manage errors through avoiding errors, trapping errors before they are committed, and mitigating the consequences of errors that are not trapped [325]. Approximately a third of CRM-based trainings include the development, redesign or implementation of learned CRM techniques/tools (e.g. briefing, debriefing, checklists) and could therefore also be categorized in this review under programme [39, 40, 42, 51, 56, 58, 59, 61, 62].

The studies show a high variety in the content of CRM training and in the results measured. The majority of the studies claim an improvement in a number of non-technical skills that were measured, but some also show that not all non-technical skills measured were improved [43, 47, 66]. Moreover, the skills that did or did not improve differed between the studies. A few studies also looked at outcome measures (e.g. clinical outcomes, error rates) and showed mixed results [49, 52, 53]. Notable is the increasing attention toward nursing CRM, which is an adaptation of CRM to nursing units [66, 67]. Most studies delivered a low to moderate quality level of evidence. Although most studies measured the effect of CRM over a longer period of time, most time periods were limited to one or two evaluations within a year. Savage et al. [58] and Ricci et al. [56] note the importance of using a longer time period.

As a result of experienced shortcomings of CRM, Team Strategies and Tools to Enhance Performance and Patient Safety (TeamSTEPPS) has evolved (since 2006). *TeamSTEPPS* is a systematic approach designed by the Agency for Healthcare Research and Quality (AHRQ) and the Department of Defense (DoD) to enhance teamwork skills that are essential to the delivery of quality and safe care. Some refer to TeamSTEPPS as “CRM and more”. TeamSTEPPS provides an approach on preparing, implementing, and sustaining team training. It is provided as a flexible training kit and facilitates in developing a tailored plan. It promotes competencies, strategies, and the use of standardized tools on five domains of teamwork: team structure, leadership, communication, situational monitoring, and mutual support. In addition, TeamSTEPPS focuses on change management, coaching, measurement, and implementation. Notable is that even though the TeamSTEPSS training is most likely to differ across settings as it needs to be tailored to the situational context, articles provide limited information on the training content. All studies report improvements in some non-technical skills (e.g. teamwork, communication, safety culture). Combining non-technical skills with outcome measures (e.g. errors, throughput time) seemed more common in this category. Half of the studies delivered a moderate to high quality of evidence.

*Simulation-based training* uses a specific method as its core, namely, simulation, which refers to “a technique to replace or amplify real-patient experiences with guided experiences, artificially contrived, that evokes or replicates substantial aspects of the real world in a fully interactive manner” [326]. The simulated scenarios that are used can have different forms (e.g. in situ simulation, in centre simulation, human actors, mannequin patients) and are built around a clinical scenario (e.g. resuscitation, bypass, trauma patients) aiming to improve technical and/or non-technical skills (e.g. interprofessional collaboration, communication). We only identified studies in a hospital setting, which were mostly focussed on an emergency setting. All studies reported improvements in some non-technical skills (e.g. teamwork behaviour, communication, shared mental model, clarity in roles and responsibilities). In addition, some studies report non-significant changes in non-technical skills [98, 137, 140, 155]. Some studies also looked at technical skills (e.g. time spend) and presented mixed results [63, 112, 152, 159]. Sixty-nine studies focused on simulation-based training, of which 16 studies delivered a moderate to high quality of evidence.

*General team training* does not focus on one specific training principle or method. It often contains multiple educational forms such as didactic lectures, interactive sessions, and online modules. General team training focuses on a broad target group and entails for example team building training, coaching training, and communication skills training. Due to the broad scope of this category, high variation in outcomes is noted, although many positive outcomes were found. Most studies have a low to very low level of evidence.

### Tools

Tools are instruments that could be implemented relatively independently in order to *structure*, *facilitate* or *trigger* teamwork.

### Structuring tools

Teamwork can be structured by using the structured communication technique SBAR (Situation, Background, Assessment, and Recommendation), (de)briefing checklists, and rounds.

*SBAR* is often studied in combination with strategies to facilitate implementation, such as didactic sessions, training, information material, and modifying SBAR material (e.g. cards) [202, 204, 206–208, 211]. In addition, this subcategory entails communication techniques similar or based on SBAR [203, 205, 209, 210, 212]. One study focused on nursing homes, while the remaining studies were performed in a hospital setting. Most studies found improvements in communication; however, a few found mixed results [208, 209]. Only (very) low-level evidence studies were identified.

Briefings and debriefings create an opportunity for professionals to systematically communicate and discuss (potential) issues before or after delivering care to a patient, based on a structured format of elements/topics or a checklist with open and/or closed-end questions. Studies on *(de)briefing checklists* often evaluate the implementation of the World Health Organization surgical safety checklist (SSC), a modified SSC, SSC-based checklist, or a safety checklist in addition to the SSC. The SSC consists of a set of questions with structured answers that should be asked and answered before induction of anaesthesia, before skin incision, and before the patient leaves the operating theatre. In addition, several studies presented checklists aiming to better manage critical events [221, 223, 233]. Only one study on SSC was conducted outside the surgery department/operating theatre (i.e. cardiac catheterization laboratory [222]). However, similar tools can also be effective in settings outside the hospital, as shown by two studies that focused on the long-term care setting [249, 260]. Overall, included studies show that (de)briefing checklists help improve a variety of non-technical skills (e.g. communication, teamwork, safety climate) and objective outcome measures (e.g. reduced complications, errors, unexpected delays, morbidity). At the same time, some studies show mixed results or are more critical of its (sustainable) effect [215, 222, 231, 242]. Whyte et al. [262] pointed out the complexity of this intervention by presenting five paradoxical findings: team briefings could mask knowledge gaps, disrupt positive communication, reinforce professional divisions, create tension, and perpetuate a problematic culture. The quality of evidence varied from high to very low (e.g. Whyte et al. [262]), and approximately one third presented a high or moderate quality of evidence. Debriefings can also be used as part of a training, aiming to provide feedback on trained skills. Consequently, some articles focused on the most suitable type of debriefing in a training setting (e.g. video-based, self-led, instructor-led) [245, 246, 253, 263] or debriefing as reflection method to enhance performance [258, 261].

*Rounds* can be described as structured interdisciplinary meetings around a patient. Rounds were solely researched in hospital settings. Five studies found improvements in non-technical skills, one study in technical skills, and one study reported outcomes but found no improvement. Three studies presented a moderate level of evidence, and the others presented a (very) low level.

### Facilitating tools

Teamwork can be facilitated through technology. Technology, such as telecommunication, facilitates teamwork as it creates the opportunity to involve and interact with professionals from a distance [271–273]. Technology also creates opportunities to exchange information through information platforms [276, 277]. Most studies found positive results for teamwork. Studies were performed in a hospital setting and presented a level of evidence varying from moderate to very low.

### Triggering tools

Teamwork could be triggered by tools that monitor and visualize information, such as (score) cards and dashboards [278, 279, 281, 283, 284]. The gathered information does not echo team performance but creates incentives for reflecting on and improving teamwork. Team processes (e.g. trust, reflection) are also triggered by sharing experiences, such as clinical cases and stories, thoughts of the day [280, 282]. All seven studies showed improvements in non-technical skills and had a very low level of evidence.

### Organizational (re)design

In contrast with the previous two categories, organizational (re)design is about changing organizational structures. Interventions can be focused on several elements within a healthcare organization, such as the payment system [292] and the physical environment [299], but are most frequently aimed at standardization of processes in pathways [286, 288] and changing roles and responsibilities [287, 289, 298], sometimes by forming dedicated teams or localizing professionals to a certain unit or patient [290, 291, 295, 300]. Most studies found some improvements of non-technical skills; however, a few found mixed results. Only four studies had a moderate level of evidence, and the others had a (very) low level.

### Programme

A programme most frequently consists of a so-called Human Resource Management bundle that combines learning and educational sessions (e.g. simulation training, congress, colloquium), often multiple tools (e.g. rounds, SBAR), and/or structural intervention (e.g. meetings, standardization). Moreover, a programme frequently takes the organizational context into account: developing an improvement plan and making choices tailored to the local situation. A specific example is the “Comprehensive Unit-Based Safety Program” (CUSP) that combines training (i.e. science of safety training educational curriculum, identify safety hazards, learn from defects) with the implementation of tools (e.g. team-based goal sheet), and structural intervention (i.e. senior executive partnership, including nurses on rounds, forming an interdisciplinary team) [309, 319, 322]. Another example is the medical team training (MTT) programme that consists of three stages: (1) preparation and follow-up, (2) learning session, (3) implementation and follow-up. MTT combines training, implementation of tools (briefings, debriefing, and other projects), and follow-up coaching [5, 304, 305, 316]. MMT programmes are typically based on CRM principles, but they distinguish themselves from the first category by extending their programme with other types of interventions. Most studies focus on the hospital setting, with the exception of the few studies performed in the primary care, mental health care, and healthcare system. Due to the wide range of programmes, the outcomes were diverse but mostly positive. The quality of evidence varied from high to very low.

## Conclusion and discussion

This systematic literature review shows that studies on improving team functioning in health care focus on four types of interventions: training, tools, organizational (re)design, and programmes. Training is divided into principle-based training (subcategories: CRM-based training and TeamSTEPPS), method-based training (simulation-based training), and general team training. Tools are instruments that could be implemented relatively independently in order to structure (subcategories: SBAR, (de)briefing checklists, and rounds), facilitate (through communication technology), or trigger teamwork (through information provision and monitoring). Organizational (re)design focuses on intervening in structures, which will consequently improve team functioning. Programmes refer to a combination of different types of interventions.

Training is the most frequently researched intervention and is most likely to be effective. The majority of the studies focused on the (acute) hospital care setting, looking at several interventions (e.g. CRM, TeamSTEPPS, simulation, SBAR, (de)briefing checklist). Long-term care settings received less attention. Most of the evaluated interventions focused on improving non-technical skills and provided evidence of improvements; objective outcome measures also received attention (e.g. errors, throughput time). Looking at the quantity and quality of evidence, principle-based training (i.e. CRM and TeamSTEPPS), simulation-based training, and (de)briefing checklist seem to provide the biggest chance of reaching the desired improvements in team functioning. In addition, programmes, in which different interventions are combined, show promising results for enhancing team functioning. The category programmes not only exemplify this trend, but are also seen in principle-based training.

Because this review is an update of our review conducted in 2008 (and published in 2010) [8], the question of how the literature evolved in the last decade arises. This current review shows that in the past 10 years significantly more research has focused on team interventions in comparison to the previous period. However, the main focus is on a few specific interventions (i.e. CRM, simulation, (de)briefing checklist). Nevertheless, an increasing number of studies are evaluating programmes in which several types of interventions are combined.

- *Training*: There has been a sharp increase in research studying team training (from 32 to 173 studies). However, the majority of these studies still look at similar instruments, namely, CRM-based and simulation-based training. TeamSTEPPS is a standardized training that has received considerable attention in the past decade. There is now a relatively strong evidence for the effectiveness of these interventions, but mostly for the (acute) hospital setting.
- *Tools*: There is also a substantial increase (from 8 to 84 studies) in studies on tools. Again, many of these studies were in the same setting (acute hospital care) and focused on two specific tools, namely, the SBAR and (de)briefing checklist. Although the level of evidence for the whole category tools is ambiguous, there is relatively strong evidence for the effectiveness of the (de)briefing checklist. Studies on tools that facilitate teamwork ascended the past decade. There is limited evidence that suggests these may enhance teamwork. The dominant setting was again hospital care, though triggering tools were also studied in other settings such as acute elderly care and clinical primary care. Moreover, most studies had a (very) low quality of evidence, which is an improvement compared to the previous review that solely presented (very) low level of evidence.
- *Organizational (re)design*: More attention is paid to organizational (re)design (from 8 to 16 studies). Although the number of studies on this subject has increased, there still remains unclarity about its effects because of the variation in interventions and the mixed nature of the results.
- *Programmes*: There seems to be new focus on a programmatic approach in which training, tools, and/or organizational (re)design are combined, often focused around the topic patient safety. The previous review identified only one such study; this research found 24 studies, not including the CRM studies for which some also use a more programmatic approach. There seems to be stronger evidence that this approach of combining interventions may be effective in improving teamwork.

### Limitations

The main limitation of this review is that we cannot claim that we have found every single study per subcategory. This would have required per subcategory an additional systematic review or an umbrella review, using additional keywords. As we identified a variety of literature reviews, future research should focus on umbrella reviews in addition to new systematic literature reviews. Note that we did find more studies per subcategory, but they did not meet our inclusion criteria. For example, we excluded multiple studies evaluating surgical checklists that did not measure its effect on team functioning but only on reported errors or morbidity. Although this review presents all relevant categories to improve team functioning in healthcare organizations, those categories are limited to team literature and are not based on related research fields such as integrated care and network medicine. Another limitation is that we excluded grey literature by only focusing on articles written in English that present empirical data and were published in peer-reviewed journals. Consequently, we might have excluded studies that present negative or non-significant effects of team interventions, and such an exclusion is also known as publication bias. In addition, the combination of the publication bias and the exclusion of grey literature has probably resulted in a main focus on standardized interventions and a limited range of alternative approaches, which does not necessarily reflect practice.

### Implication for future research

This review shows the major increase in the last decade in the number of studies on how to improve team functioning in healthcare organizations. At the same time, it shows that this research tends to focus around certain interventions, settings, and outcomes. This helped to provide more evidence but also left four major gaps in the current literature. First, less evidence is available about interventions to improve team functioning outside the hospital setting (e.g. primary care, youth care, mental health care, care for disabled people). With the worldwide trend to provide more care at home, this is an important gap. Thereby, team characteristics across healthcare settings vary significantly, which challenges the generalizability [327]. Second, little is known about the long-term effects of the implemented interventions. We call for more research that monitors the effects over a longer period of time and provides insights into factors that influence their sustainability. Third, studies often provide too little information about the context. To truly understand why a team intervention affects performance and to be able to replicate the effect (by researchers and practitioners), detailed information is required related to the implementation process of the intervention and the context. Fourth, the total picture of relevant outcomes is missing. We encourage research that includes less frequently used outcomes such as well-being of professionals and focuses on identifying possible deadly combinations between outcomes.

## Supplementary information

**Additional file 1.** Search syntax EMBASE (DOCX 12 kb)

**Additional file 2.** GRADE (DOCX 13 kb)

## Data Availability

Not applicable

## Abbreviations

AHRQ: Agency for Healthcare Research and Quality
CRM: Crew resource management
CUSP: Comprehensive Unit-Based Safety Program
DoD: Department of Defense
GRADE: Grading of Recommendations Assessment Development, and Evaluation
MTT: Medical team training
NOTECHS: Non-Technical Skills
PRISMA: Preferred Reporting Items for Systematic Reviews and Meta-Analyses
SAQ: Safety Attitude Questionnaire
SBAR: Situation, Background, Assessment, and Recommendation
SSC: Surgical safety checklist
TeamSTEPPS: Team Strategies and Tools to Enhance Performance and Patient Safety
