# Supplementary material for: Interventions to improve team effectiveness within health care: a systematic review of the past decade
Source: Hum Resour Health. 2020 Jan 8;18:2. doi: 10.1186/s12960-019-0411-3 (PMC6950792; doi:10.1186/s12960-019-0411-3)
Supplement: Supplementary file 1 — Additional file 1. Search syntax EMBASE (DOCX 12 kb) [file 12960_2019_411_MOESM1_ESM.docx]

**Additional file 1 Search syntax Embase**

('team building'/de OR 'teamwork'/de OR (teamwork* OR teambuilding* OR (team* NEAR/3 (work OR building OR program*))):ab,ti) AND ('total quality management'/de OR (((qualit* OR team* OR safet* OR innovation* OR effectiv* OR healthcare OR care OR outcome* OR perform* OR function* OR staff* OR communicat* OR awarenes* OR skill* OR efficien* OR productiv* OR leadership* OR knowledge* OR information* OR task* OR attitude* OR culture* OR climate* OR collaborat* OR behav* OR error* OR workload OR learn* OR efficac* OR cohesi* OR enjoy* OR turnover OR turn-over OR patient* OR process* OR satisf* OR involve* OR relation* OR retention* OR absen* OR sick-leave OR length-of-stay OR resilien* OR well-being OR wellbeing OR innovat* OR competence*) NEAR/6 (improv* OR enhanc* OR increas* OR decrease* OR reduc*))):ab,ti) AND ('program evaluation'/exp OR 'intervention study'/de OR 'education'/de OR (program* OR intervention* OR education* OR training OR tool OR tools OR checklist*):ab,ti) AND ('health care personnel'/exp OR (health OR care OR healthcare OR nurse* OR medical* OR hospital* OR clinic* OR doctor* OR surg* OR Physician* OR psychiatr* OR paramedic*):ab,ti) NOT ([Conference Abstract]/lim OR [Letter]/lim OR [Note]/lim OR [Editorial]/lim) AND [english]/lim
