# Supplementary material for: Interventions to improve team effectiveness within health care: a systematic review of the past decade
Source: Hum Resour Health. 2020 Jan 8;18:2. doi: 10.1186/s12960-019-0411-3 (PMC6950792; doi:10.1186/s12960-019-0411-3)
Supplement: Supplementary file 2 — Additional file 2. GRADE (DOCX 13 kb) [file 12960_2019_411_MOESM2_ESM.docx]

**Additional file 2 GRADE**

| **GRADE** | **Example of study designs** |
| --- | --- |
| A high quality of evidence | multicenter RCT, large high-quality multi-center trial, high-quality pre- and post surveys |
| B moderate quality of evidence | one-center RCT, RCT with severe limitations, and pre-and post surveys |
| C low quality of evidence | high-quality qualitative studies, quasi-experimental designs and pre-and post surveys with limitations |
| D very low quality of evidence | low- quality qualitative studies and pre- and post surveys with severe limitations |
| **Decrease grade if:**   - Serious ( − 1) or very serious ( − 2) limitation to study quality - Important inconsistency ( − 1) - Some ( − 1) or major ( − 2) uncertainty about directness - Imprecise or sparse data ( − 1) - High probability of reporting bias ( − 1) | |
| **Increase grade if:**   - Strong evidence of association—significant relative risk of > 2 ( < 0.5) based on consistent evidence from two or more observational studies, with no plausible confounders (+1) - Very strong evidence of association—significant relative risk of > 5 ( < 0.2) based on direct evidence with no major threats to validity (+2) - Evidence of a dose response gradient (+1) - All plausible confounders would have reduced the effect (+1) | |
